# Pathogenic genetic variants from highly connected cancer susceptibility genes confer the loss of structural stability

**DOI:** 10.1038/s41598-021-98547-y

**Published:** 2021-09-28

**Authors:** Mahjerin Nasrin Reza, Nadim Ferdous, Md. Tabassum Hossain Emon, Md. Shariful Islam, A. K. M. Mohiuddin, Mohammad Uzzal Hossain

**Affiliations:** 1 Department of Biotechnology and Genetic Engineering, Faculty of Life Science, Mawlana Bhashani Science and Technology University, Santosh, Tangail, 1902 Bangladesh; 2 Department of Biology, University of Kentucky, 101 T.H. Morgan Building, Lexington, KY 40506-022 USA; 3 Bioinformatics Division, Ministry of Science and Technology, National Institute of Biotechnology, Ganakbari, Ashulia, Savar, Dhaka, 1349 Bangladesh

**Keywords:** Cancer, Computational biology and bioinformatics

## Abstract

Genetic polymorphisms in DNA damage repair and tumor suppressor genes have been associated with increasing the risk of several types of cancer. Analyses of putative functional single nucleotide polymorphisms (SNP) in such genes can greatly improve human health by guiding choice of therapeutics. In this study, we selected nine genes responsible for various cancer types for gene enrichment analysis and found that BRCA1, ATM, and TP53 were more enriched in connectivity. Therefore, we used different computational algorithms to classify the nonsynonymous SNPs which are deleterious to the structure and/or function of these three proteins. The present study showed that the major pathogenic variants (V1687G and V1736G of BRCA1, I2865T and V2906A of ATM, V216G and L194H of TP53) might have a greater impact on the destabilization of the proteins. To stabilize the high-risk SNPs, we performed mutation site-specific molecular docking analysis and validated using molecular dynamics (MD) simulation and molecular mechanics/Poisson Boltzmann surface area (MM/PBSA) studies. Additionally, SNPs of untranslated regions of these genes affecting miRNA binding were characterized. Hence, this study will assist in developing precision medicines for cancer types related to these polymorphisms.

## Introduction

Now-a-days, the number of cancer incidence has been increasing globally. Genetic factors play a significant role in the development of a majority of cancers as in these cases, genes regulating cell division, apoptosis, invasiveness, or metastasis undergo mutation. Several important cancers such as breast, ovarian, esophageal, lung, colon, colorectal, melanoma, pancreatic cancers, etc. develop due to the accumulation of mutations in BRCA1^1^, BRCA2^1^, ATM^2^, TP53^3^, MSH2^4^, MLH1^4^, MSH6^5^, CDKN2A^6^, and PALB2^7^ genes. Mutations in the BRCA1 and the BRCA2 genes are responsible for 90% of hereditary breast cancer and the majority of hereditary ovarian cancer cases^1,8–10^. The ATM, BRCA1, BRCA2, and TP53 are known as “caretaker” genes involved in DNA repair and function in the maintenance of genomic stability^2^. Mutations in ATM lead to T-cell prolymphocytic leukemia, B-cell chronic lymphocytic leukemia and it was also observed at increased frequency in breast cancer cases^2^. Somatic TP53 mutations occur in ovarian, esophageal, colorectal, head and neck, larynx, and lung cancers at rates ranging from 38 to 50%^3^. MLH1 and MSH2 mutations are known to be associated with lifetime ovarian and endometrial cancer^4^. Increased risk of colorectal carcinoma occurs in the MSH6 mutation carriers^5^. Germline alterations in CDKN2A have been identified in melanoma cases^6^. Apart from mutations in major genes responsible for breast cancer, germline mutations in PALB2 have been identified in familial breast cancer and familial pancreatic cancer cases^7^. Insights into the molecular mechanisms underlying the effects of mutations that result in cancer susceptibility are indispensable considering their crucial roles in cell cycle regulation, metabolism, DNA mismatch repair, and immunity.

Single nucleotide polymorphisms (SNPs) are the most frequent type of genetic alteration in humans consisting of about 90% of sequence variants^11^. They serve as indicators in the detection of part of the genome involved in disease^12^. They are dispersed throughout the genome in both coding and regulatory regions of genes and the most important SNPs are the ones that are in the coding region of the human genome consisting of around 500,000 SNPs^13^. Among these, missense SNPs, also known as non-synonymous SNPs (nsSNPs) are especially significant as they are responsible for amino acid substitutions resulting in structural and functional variations of proteins^14^. Genetic tools and comparative genomics have been used for large-scale extraction of SNPs over the years. More than 10 million SNPs have been identified covering the most common polymorphisms^15^. Currently, the most common polymorphisms that have been identified covers more than 10 million SNPs^15^. DNA sequencing is the first step towards understanding the nature of a variation^16^. With the advancement of computational algorithms and in silico methodologies, cost-effective, robust, and refined methods are being created, helping not only in mutational studies, but also impacted the entire drug and vaccine development trajectory, discovering new potential lead candidates with a remarkable reduction to cost and time^17–20^. The progress has also aided in the development of high-throughput tools for the identification of structural and dynamic changes of protein products as a result of SNPs^21,22^. Although most of the SNPs cause damages to the protein, some of them are neutral^23^. Therefore, a proper selection of computational methods and algorithms are required for the prediction of structural and functional consequences of the target proteins to distinguish the deleterious nsSNPs from the neutral ones.

Most of the proteins carry out functions primarily through their integral domains. These are independent units having potentially different biological functions and they can be gained by proteins in order to acquire novel functions^24^. Domains are, therefore, defined to be the functional units through which proteins evolve. Numerous computational studies have been conducted to analyze the nsSNPs of cancer susceptibility genes. A study analyzed genetic variations in the BRCA1-associated RING domain protein encoded by the BARD1 gene and predicted their deleterious effects causing breast, ovarian and uterine cancers^25^. Similar study identified missense SNPs in the homeobox domain protein encoded by the human HOXB13 gene which is responsible for hereditary prostate cancer^26^. The nsSNPs in the RASSF5 gene that plays a crucial role as a tumor suppressor were found to reduce the binding affinity with H-Ras protein^27^. As nsSNPs result in amino acid substitutions of a protein, presence of these polymorphisms in domains alters protein interactions, functions and post-translational modifications^28^. But majority of these studies focus on nsSNPs located on regions other than the significant protein domains. All the analyses were performed on a single gene and there was no connection shown with other cancer susceptibility genes in these studies. Also, there were no further experiments conducted to stabilize these deleterious nsSNPs keeping a gap in identifying the choice of probable therapeutics.

In this study, we conducted our investigation to identify and evaluate the pathogenic nsSNPs in highly connected cancer susceptibility genes, their associations in causing diseases, and the effect of these deleterious nsSNPs in the structural behavior of proteins. Several computational algorithms were merged to address these pathogenic mutations. In order to scrutinize the rescue mechanisms of the damaging substitutions, we performed mutation site targeted protein–ligand docking with a carbazole derivative, PhiKan083 (PK083). Molecular dynamics simulation was performed for clear depiction of the dynamic behavior of WT and mutants over time. Finally, we identified the functional impact of 3′ and 5′ SNPs in the binding of miRNA.

## Results

### Gene enrichment

**Figure 1:**
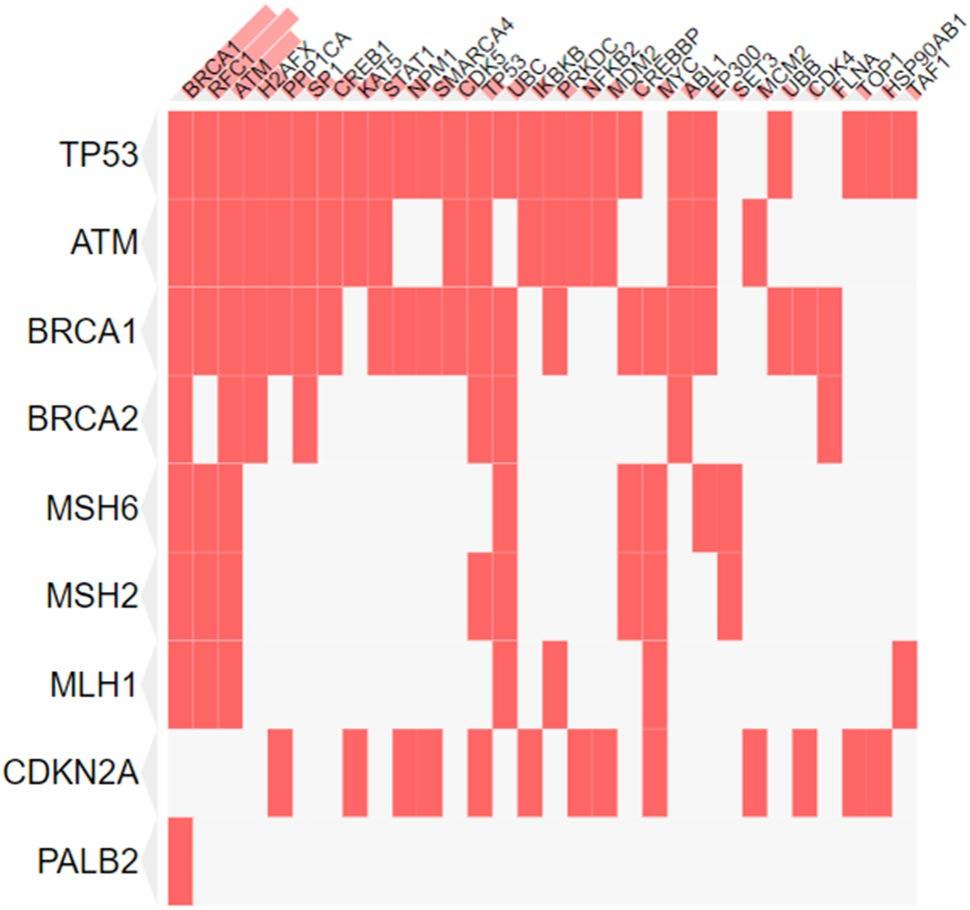
Gene enrichment result from the Enrichr server. From the curated protein–protein interactions, BRCA1, ATM and TP53 is more enriched in connectivity on a clustegram view.

From the Enrichr result, we found that BRCA1 and ATM seem to have high likelihood and greater degree of interaction than other genes (Fig. 1). The P-value was computed from the fisher’s exact test, a proportion test assuming binomial distribution and independence of probability of any gene belonging to any set (Table 1). Based on the enrichment analysis and P-value, we selected BRCA1, ATM, and TP53 gene for analysis of SNPs.

**Table 1:** The p-value of genes computed from the fisher’s exact test.

Gene term	P-value	Adjusted P-value	Odds ratio
BRCA1	1.45E−15	5.58E−13	82.30452675
ATM	6.67E−13	1.28E−10	70.07007007
TP53	1.91E−08	1.84E−06	26.56042497

### SNP datasets retrieved from dbSNP database

**Figure 2:**
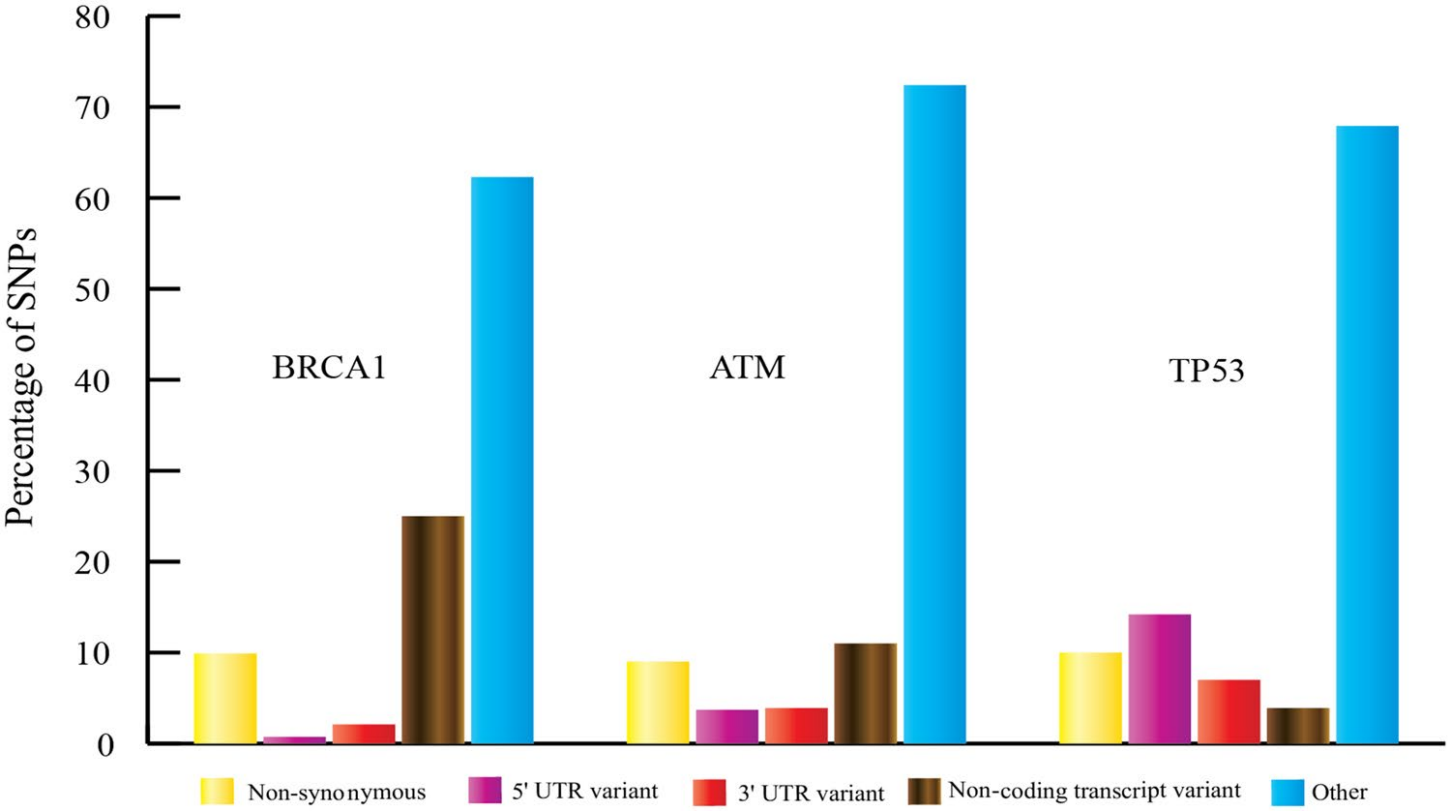
Bar diagram showing the percentages of the SNPs in BRCA1, ATM and TP53 genes. BRCA1 gene had 9.9% nsSNPs, 0.72% 5′ UTR SNPs, 2.1% 3′ UTR SNPs, 9% non-coding transcript variants and 62.28% other SNPs of total SNPs; ATM gene had 9% nsSNPs, 3.7% 5′ UTR SNPs, 3.9% 3′ UTR SNPs, 11% non-coding transcript variants and 72.4% other SNPs of total SNPs; TP53 gene had 10% nsSNPs, 14.2% 5′ UTR SNPs, 7% 3′ UTR SNPs, 3.9% non-coding transcript variants and 67.9% other SNPs of total SNPs.

We retrieved the SNPs of BRCA1, ATM and TP53 genes from dbSNP database. We found a total number of 25,754 SNPs of BRCA1, 41,948 SNPs of ATM and 6977 SNPs of TP53 in the dbSNP database. The percentage of different types of SNPs of each gene are shown in Fig. 2. We selected only the nsSNPs of the three genes for our investigation. Finally, including the multiple allele changes, we have analyzed a total number of 3467 nsSNPs of BRCA1, 4650 nsSNPs of ATM and 1106 nsSNPs of TP53 for our investigation.

### Deleterious and damaging nsSNPs in BRCA1, ATM and TP53

We subjected all the nsSNPs of the three genes to SIFT, PolyPhen-2 and PROVEAN tools to investigate the effect of amino acid substitution on the respective protein function. We shortlisted those nsSNPs as highly deleterious that were predicted damaging/probably damaging or deleterious by at least two of these tools. Total 1013 nsSNPs of BRCA1, 1815 nsSNPs of ATM and 528 nsSNPs of met the criteria and we classified them as highly deleterious nsSNPs. Prediction results by SIFT, PolyPhen2 and PROVEAN are shown in Supplementary File 1–3.

### Disease-associated nsSNPs in BRCA1, ATM, and TP53

The above shortlisted nsSNPs were submitted to PhD-SNP, PANTHER, SNPs&GO, SNAP2, and PredictSNP tools to identify the disease-associated nsSNPs. We shortlisted the nsSNPs those were found deleterious by all the five tools. A total number of 250 nsSNPs of BRCA1, 796 nsSNPs of ATM, and 341 nsSNPs of TP53 were predicted deleterious by all the five tools and were considered for further investigation (Supplementary File 4–6).

### Identified nsSNPs in conserved domains

**Figure 3:**
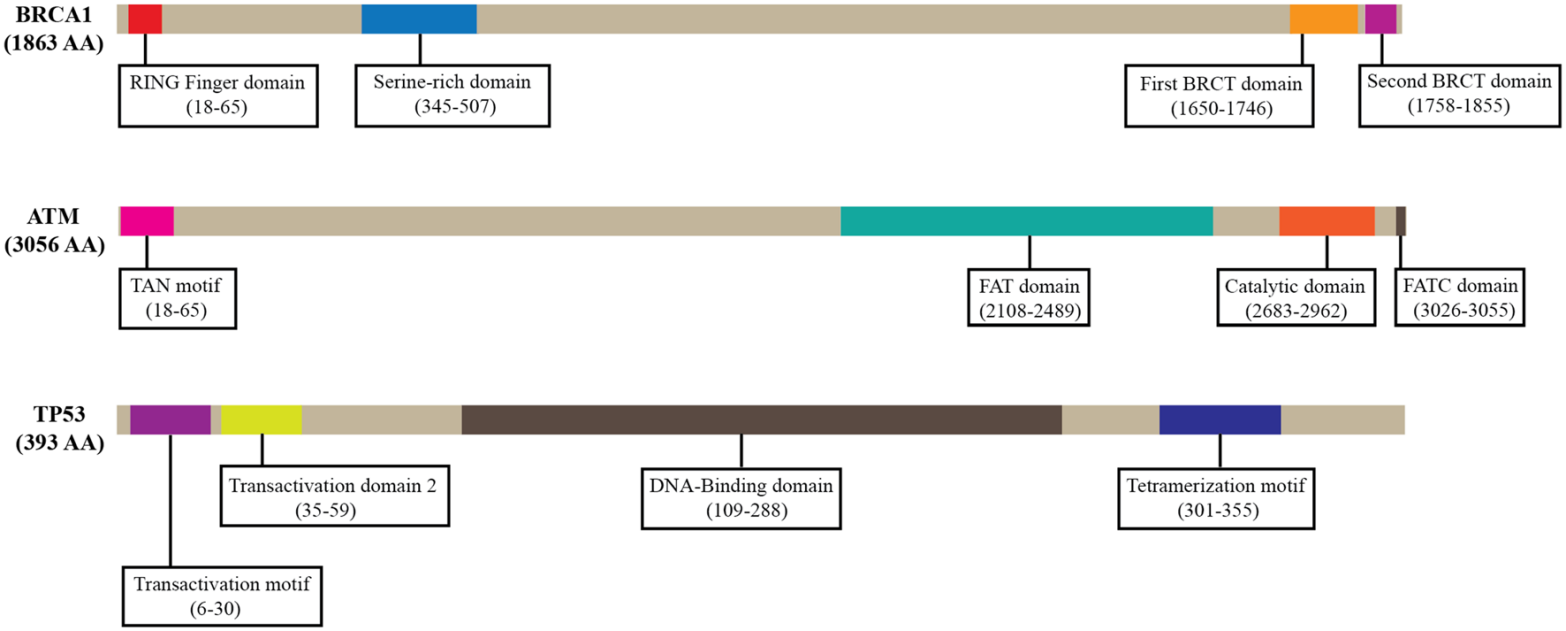
Domains and sequence intervals of BRCA1, ATM and TP53 proteins.

NCBI’s conserved domain search tool revealed that each of the BRCA1, ATM, and TP53 proteins were found to have four domains shown in Fig. 3. Results showed that the BRCA1 protein had a serine-rich domain associated with BRCT, the first BRCT domain, the second (C-terminal) BRCT domain and a RING finger domain. Originally BRCT domain was identified in the tumor suppressor protein and missense mutations in this region correspond to a high risk for breast and ovarian cancers^9,29^. The ATM protein was found to have a catalytic domain, a FAT domain, a TAN domain, and a FATC domain. The catalytic domain of ATM is pivotal for phosphorylation of dozens of substrates that are involved in repair of DNA double strand breaks^30^. The FAT domain is located adjacent and upstream of the kinase domain and the name of this domain is derived from FRAP (mTOR), ATM, and TRAPP, all of which members of the phosphoinositide 3-kinase-like family^31^. ATM protein also contains a Tel1/ATM N-Terminal Motif (TAN) that is essential for telomere length maintenance and DNA damage response^32^. Search results of p53 protein showed that it had a p53 DNA-binding domain (DBD), a p53 tetramerization motif, the transactivation domain 2 (TAD2), and a p53 transactivation motif. The DNA-binding domain is absent in p63, p73 and other p53 homologues in primitive organisms thus making it a unique feature for the vertebrate p53^33^. A flexible linker region connects the structured DNA-binding and tetramerization domains of p53^34^. There are 2 distinct TADs found in p53 and it was found that TAD2 also play important part in tumor suppression^35^. As domains play significant role in proteins, we shortlisted the disease-associated nsSNPs that fall on the domain sequences of the three proteins. From CD-search results, we found that, 171 nsSNPs of BRCA1, 313 nsSNPs of ATM and all the shortlisted 340 nsSNPs of TP53 occur on the predicted domains and were considered for further analysis (Supplementary File 7–9).

### Conservation profile of deleterious nsSNPs in BRCA1, ATM and TP53

We calculated the evolutionary conservation of amino acid residues of the three proteins using ConSurf server to further explore the possible effects of shortlisted nsSNPs. Results were collected in the form of structural representation of the protein sequence. Based on the location either on protein surface or inside its core, the highly conserved residues are predicted as either functional or structural. Amino acids involved in various vital biological processes appear to be more conserved than others. Considering this, the nsSNPs located at these conserved regions are highly damaging to proteins as compared to those located at non-conserved sites^36,37^. Hence, we focused only on the residues of domains matching their positions with the shortlisted high risk nsSNPs. The results predicted that out of the shortlisted nsSNPs, 89 nsSNPs of BRCA1, 218 nsSNPs of ATM and 282 nsSNPs of TP53 were highly conserved (either exposed or buried). Supplementary File 10–12 contains the graphical representation of ConSurf results of the three proteins.

### Predicted stability modification

We analyzed the stability alterations in the three proteins using I-Mutant server which completed this task by considering the amino acid substitutions. I-mutant 2.0 results revealed that all the 89 nsSNPs of BRCA1, 192 nsSNPs of ATM, 245 nsSNPs of TP53 decrease stability of the respective proteins (Supplementary File 13–15). Thus, these polymorphisms in the protein domains might cause supreme damage to the protein affecting their stability. According to some studies, phenomenon such as increase in degradation, misfolding and aggregation of proteins are caused by decreased protein stability^38–40^. So, considering the lower ΔΔG values, we finally selected top five nsSNPs (Table 2) from each protein and considered them for 3D structure modeling.

**Table 2:** I-MUTANT 2.0 predictions for nsSNPs with lower DDG values in BRCA1, ATM and TP53.

Gene	nsSNP ID	Amino acid change	Stability	DDG
BRCA1	rs45553935	V1736G	Decrease	− 2.7
	rs80357107	V1838G	Decrease	− 2.37
	rs80357451	V1832G	Decrease	− 2.32
	rs1555579627	V1687G	Decrease	− 2.3
	rs730881496	L1729Q	Decrease	− 2.25
ATM	rs201216427	V2757G	Decrease	− 2.81
	rs587779873	I2865T	Decrease	− 2.47
	rs876659516	I2948T	Decrease	− 2.29
	rs1591264625	V2830G	Decrease	− 2.12
	rs730881328	V2906A	Decrease	− 2.1
TP53	rs1057520004	V216G	Decrease	− 2.56
	rs1057519998	L194H	Decrease	− 2.51
	rs1330865474	I254S	Decrease	− 2.48
	rs730882027	I251T	Decrease	− 2.34
	rs760043106	I195T	Decrease	− 2.28

### Characterized functional effects of SNPs in 3′ and 5′ untranslated regions

PolymiRTS database predicted the list of miRNAs disrupted and created by SNPs of ATM and TP53 genes. Interestingly, no miRNAs were found to be disrupted or created by BRCA1 gene from the database. Supplementary File 16 contains the tables (Supplementary Tables S1–S4) showing the effect of SNPs in the 3′ and 5′ region of ATM and TP53 genes. Higher conservation score indicates greater effect of the SNPs. In addition, higher context + score denotes higher likelihood of disruption or creation that occurs in the miRNA target site. The miRNAs greatly affected by the SNPs of ATM and TP53 genes based on highest conservation score are shown in Table 3.

**Table 3:** Target sites disrupted and created by single nucleotide polymorphisms (SNPs) in miRNA seeds.

Gene	SNP ID	miR ID	miRSite	Conservation	Context + score change
**Target sites disrupted by SNPs and INDELs in miRNA seeds**					
ATM	rs3745198	hsa-miR-6796-3p	AGAGCUU	5	− 0.025
TP53	rs71309450	hsa-miR-1233-5p	UCCCACA	5	− 0.192
**Target sites created by SNPs and INDELs in miRNA seeds**					
ATM	rs190453265	hsa-miR-525-3p	GCACCUUA	7	− 0.438
TP53	rs201549145	hsa-miR-548i	UUAUUUUA	11	0.014

### Structural insights into BRCA1, ATM, p53 domains and their mutants

**Figure 4:**
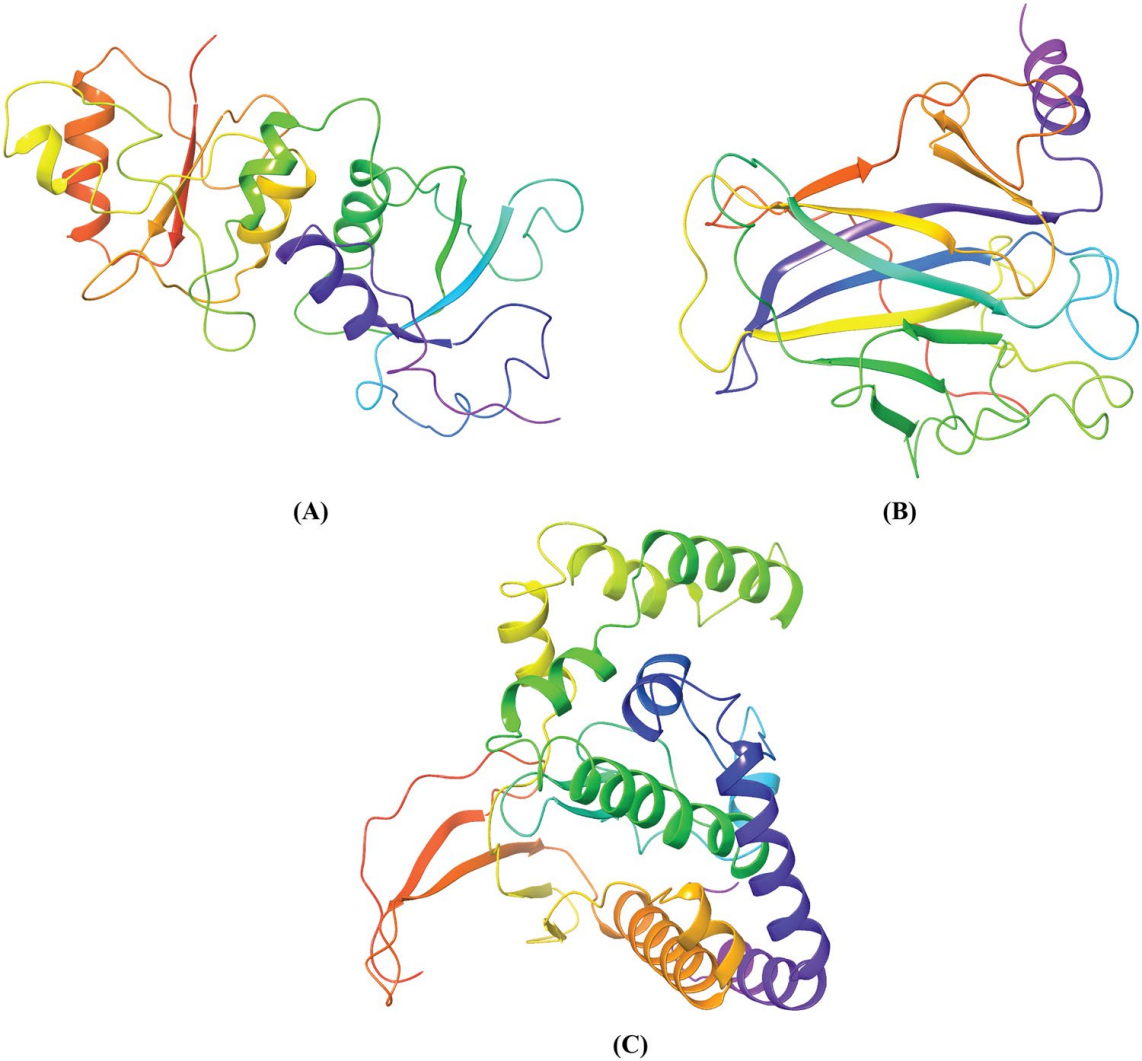
3D structures of three domains from BRCA1, ATM and p53 protein. (**A**) Crystal structure of BRCT domain of BRCA1 at 3.50 Å resolution. (**B**) DNA binding domain of p53 at 1.92 Å resolution. (**C**) Modelled 3D structure of the catalytic domain of ATM. We used 5NP0 as template to model the domain.

The shortlisted 15 nsSNPs of BRCA1, ATM and TP53 were taken in order to the 3D structures of mutated domains. All the nsSNPs of BRCA1 fall on the first BRCT domain of BRCA1 protein. The shortlisted nsSNPs of ATM and TP53 fall on the catalytic domain and the DNA-binding domain of the two proteins respectively. The crystal structure of BRCA1 BRCT (PDB ID: 4JLU) and p53 DNA binding domain (PDB ID: 2PCX) were available in the RCSB PDB database. These two structures were retrieved and cleaned by removing the ligands and inhibitors (Fig. 4). The “Mutagenesis” tool of Pymol was utilized to carry out mutation with the selected five nsSNPs of each protein. The 3D structure of the catalytic domain of ATM was not available in PDB database. So, the structure was modeled through MODELLER 9.22 using the 3D structure of closed dimer of human ATM solved by electron microscopy at 5.70 Å resolution^41^ (PDB ID: 5NP0) and was later refined using GalaxyRefine server. The Ramachandran plot analysis, ERRAT server and ProSA-Web analysis results of the refined structure are shown in Supplementary File 16 (Supplementary Fig. S1). Later the structural analysis was extended by calculating the RMSD values for each mutant model using Maestro 11.8 tool of the Schrodinger suite. The average distance among all atoms, α-carbon atoms and backbones of WT and mutant models were measured from RMSD values. Greater RMSD value indicates greater deviation of mutant structures from that of the WT proteins. To further validate the mutation inducing method, we carried out homology modeling of all the 15 mutants from 3 proteins though MODELLER 9.22 using the same templates as stated earlier and upon performing refinement using GalaxyRefine server, we calculated their RMSD values. Both the Pymol generated mutant models and homology modeled mutant structures for V1687G and V1736G of BRCT, I2865T and V2906A of Catalytic domain, V216G and L194H of p53 exhibited the maximum RMSD values shown in Table 4. From the Stride server results, it was found that V1687G and V216G mutations were in β-strand region, V1736G, V2906A and L194H mutations were in coil region and I2865T mutation was in α-helix region of the respected proteins (Supplementary Fig. S2). Also, majority of the mutations caused significant increase in solvent accessible area (Supplementary Table S5). Analysis from ProtParam server revealed that all the mutants had lower instability index and aliphatic index than the WT proteins (Supplementary Table S6).

**Table 4:** Predicted RMSD (All atoms, Cα atoms and Backbone) values of WT and mutants of BRCA1, ATM and TP53 calculated on both mutated proteins and modeled mutants.

Gene	SNP ID	Amino acid substitutions	Proteins mutated using Pymol			Proteins modeled using MODELLER		
			RMSD (all atoms)	RMSD (Cα atoms)	RMSD (backbone)	RMSD (all atoms)	RMSD (Cα atoms)	RMSD (backbone)
BRCA1	rs45553935	V1736G	3.3315	0.0019	0.0012	4.0309	0.2657	0.3463
	rs80357107	V1838G	1.4924	0.0014	0.0015	3.2530	0.2563	0.3345
	rs80357451	V1832G	1.6726	0.0005	0.0015	3.3261	0.2841	0.3454
	rs1555579627	V1687G	3.9798	0.0017	0.0016	4.3966	0.2512	0.3728
	rs730881496	L1729Q	2.0206	0.0018	0.0015	2.7943	0.2905	0.3546
ATM	rs201216427	V2757G	2.6360	0.0015	2.0706	2.2624	0.9240	0.9148
	rs587779873	I2865T	3.7960	0.0011	1.9320	3.4941	0.9002	0.8567
	rs876659516	I2948T	3.1163	0.0012	2.0694	1.8218	0.7576	0.7340
	rs1591264625	V2830G	2.7638	0.0020	2.0559	2.1558	0.6263	0.5973
	rs1555053927	V2906A	3.1687	0.0010	1.9769	3.1090	1.0781	1.0348
TP53	rs1057520004	V216G	2.6572	0.0020	0.0017	3.7278	0.2980	0.3848
	rs1057519998	L194H	2.5904	0.0022	0.0019	3.3840	0.1719	0.2557
	rs1330865474	I254S	1.6255	0.0023	0.0014	3.3189	0.2008	0.2592
	rs730882027	I251T	1.2445	0.0018	0.0018	3.2160	0.2536	0.3008
	rs760043106	I195T	1.8478	0.0014	0.0017	2.5908	0.2767	0.3095

### Stabilization of high-risk mutants by PK083

**Figure 5:**
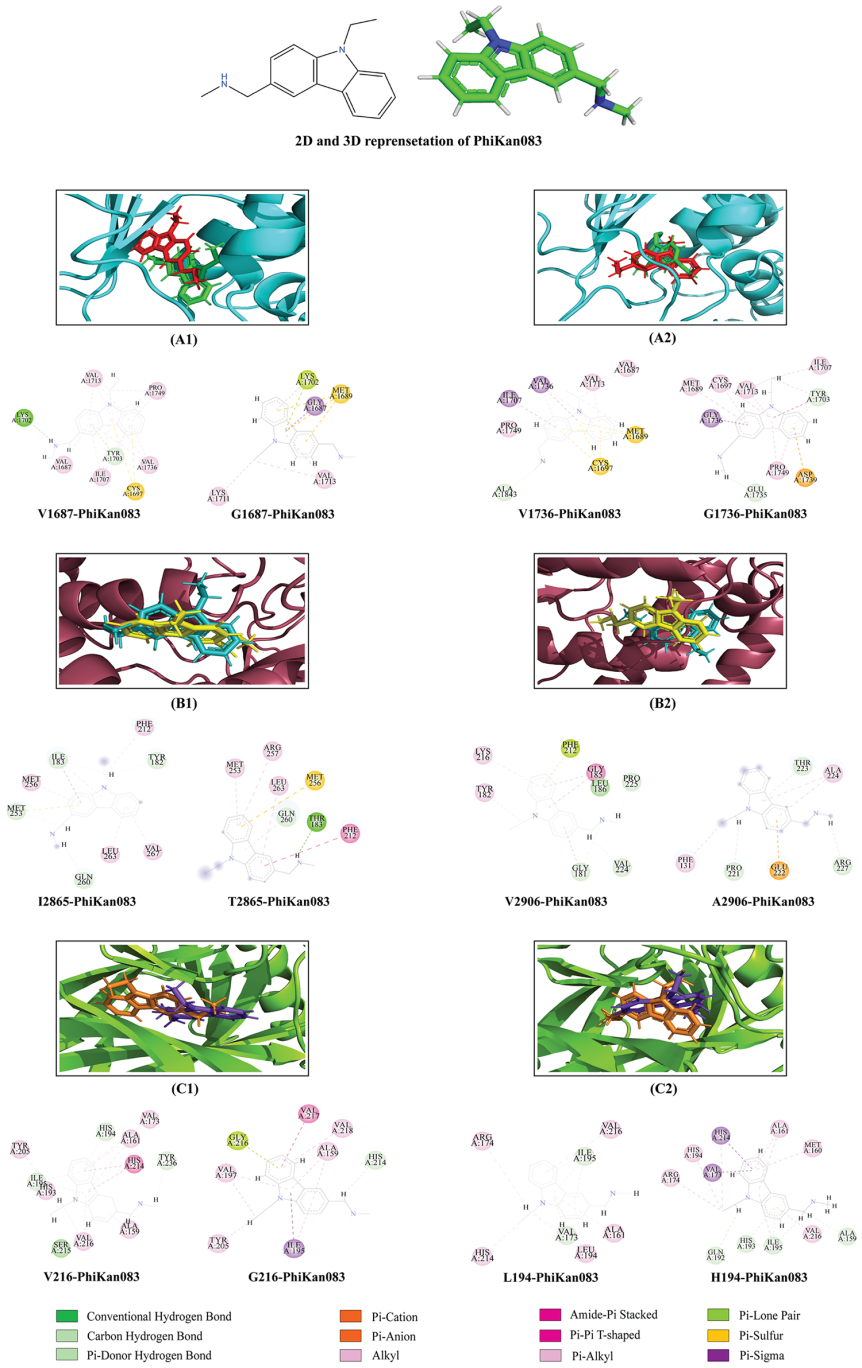
Structure of PhiKan083 and interactions with WT proteins and mutants. The 2D and energy minimized 3D structure of (**A**) PK083. Superimposed images of bonded PK083 docked against WT and mutant proteins in the mutation location are shown here. (**A1,A2**) PK083 bonded to WT (light green) and mutant (red) BRCT domains. (**B1,B2**) PK083 bonded to WT (cyan) and mutant (yellow) catalytic domains. (**C1,C2**) PK083 bonded to WT (purple) and mutant (orange) DNA-binding domains.

PK083 belongs to a class of organic compounds known as carbazoles and they contain a three-ring system containing a pyrrole ring fused on either side to a benzene ring. Several carbazole derivatives such as PK083, PK9284, PK9295, PK9318, PK9320 etc. were tested to stabilize Y220S and Y220N p53 mutants and their binding constant (Kd) were also determined^42^. Among them, PK083 (PhiKan083), was found to bind to the cavity formed due to mutation with a dissociation constant of ≈ 150 μM50^42^. It was also found to raise the melting temperature of the mutant and to slow down its rate of thermal denaturation. The crystal structure of the protein–PK083 complex at 1.5-Å resolution (PDB ID: 2VUK) was solved and available in the PDB database. From molecular interaction analysis we found that PK083 forms a pi-sulfur bond with the mutant Cys220 residue. As BRCA1, ATM and TP53 were found to be more enriched in connectivity, we hypothesized that, PK083 might stabilize the most damaging mutations of these three proteins. Previously, in-silico investigation was performed to identify the stabilization capability of PK083 to other mutants of Y220^43^. From the study, it was found that Y220S showed a similar interaction pattern with PK083. The study also showed that structural optimization of PK083 might possibly lead to a novel drug that can interact favorably with another mutant, Y220N^43^. The Y220H and other mutants of p53 including the WT protein were not found to favorably interact with PK083. Molecular docking of PK083 with six mutants as well as WT target sites exhibited nearly same score in binding affinity. The binding affinity of this molecule with the mutants ranges from − 21.1 to − 99.5 kcal/mol shown in Table 5. PK083 binds at the binding pockets of mutation positions as the defined binding sites of three proteins. Highest number of interactions was observed in L194H-PK083 complex where PK083 was bonded to the mutant residue with a Pi-alkyl bond. Interaction of PK08 with the mutants were found to be largely hydrophobic (Fig. 5). It was also observed that Val1713 was common participant residue in the BRCT mutant-PK083 interactions while Ala159 and Ile195 were found to be common interacting residues in the DBD mutant-PK083 complexes. Pi-Sigma, Pi-Alkyl, Pi-Lone Pair and conventional hydrogen bonding interactions were observed in the binding of PK083 with the mutant residues. The common residues of interactions in WT and mutants with PK083 are shown in Supplementary Table S7.

**Table 5:** Interactions of PK083 with six mutants along with the WT proteins of BRCA1 (BRCT domain), ATM (Catalytic domain) and p53 (DNA-Binding domain).

Protein	WT/mutations	Binding score (kcal/mol)	Interacting bond with WT/mutant residue	Distance (Å)
BRCA1	WT-V1687	− 77.6	Pi-alkyl	4.66
	V1687G	− 68.2	Pi-sigma	2.02
	WT-V1736	− 76.81	Pi-alkyl	5.41
	V1736A	− 65.7	Pi-sigma	2.09
ATM	WT-I183	− 75.6	Carbon hydrogen	3.09
	I183T	− 72.3	Hydrogen	1.75
	WT-V224	− 99.5	Carbon hydrogen	2.86
	V224A	− 66.3	Pi-alkyl	4.44
TP53	WT-L194	− 24.9	Pi-alkyl	4.05
	L194H	− 32.2	Pi-alkyl	5.36
	WT-V216	− 21.1	Pi-alkyl	4.59
	V216G	− 32.8	Pi-lone pair	2.83

### Molecular dynamics (MD) simulation

**Figure 6:**
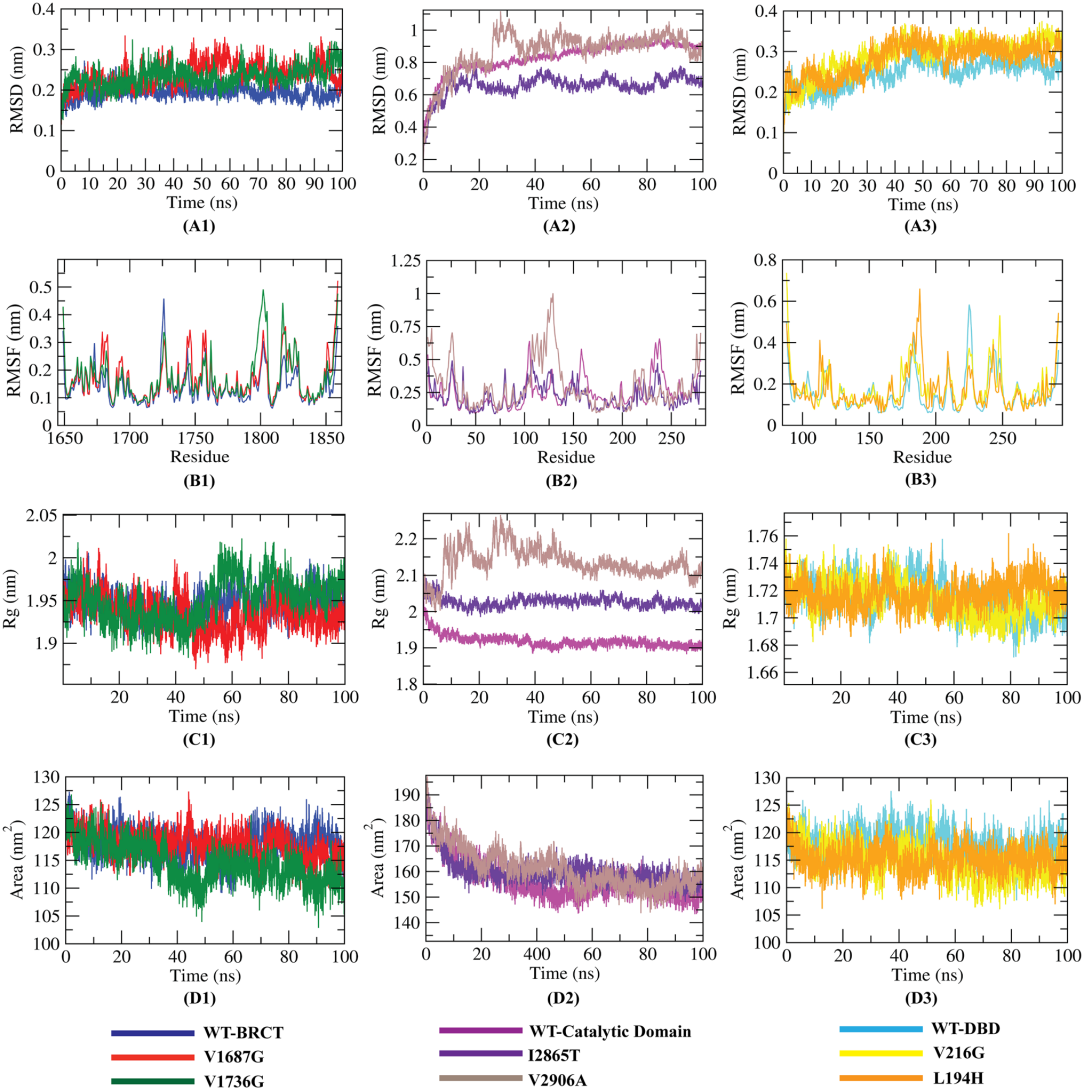
MD simulation results (1st independent run) of WT and mutant variants. (**A1–A3**) RMSD analysis, (**B1–B3**) RMSF analysis, (**C1–C3**) radius of gyration (Rg) analysis, and (**D1–D3**) SASA analysis.

As physiological conditions are not considered in evaluating the damaging nature of the mutants using computational tools, we performed MD simulation of both the WT protein domains and the six mutants to view the various conformations that they might acquire in the solvated state. Their dynamic behavior was analyzed by RMSD, RMSF, Rg, and SASA analysis. The analyses from 1st independent MD run are presented in Fig. 6 while analyses from 2nd are 3rd MD run is shown in Supplementary Figs. S3 and S4 respectively. Table 7 contains the average values obtained from trajectory analyses of 3 independent production runs. The number of solvent molecules, Na, and Cl ions added to each system are shown in Supplementary File 16 (Supplementary Table S8).

Configuration changes of all the WT and mutant proteins were analyzed in terms of RMSD during the simulation period. Figure 6(A1,A2) depicts that RMSD values from the mutant structures are quite unstable comparing with the WT-BRCT and WT-DBD. The WT BRCT shows steady fluctuation throughout the 100 ns in the WT structure. V1687G and V1736G structures showed similar way of deviation till 45 ns from their starting structure, after that, fluctuated up to ~ 0.3 nm for V1687G. RMSD values of V216G and L194H were higher than the WT protein’s RMSD at major points demonstrating that the mutations have considerable destabilizing effects on DBD. Interestingly, RMSD values of I2865T were lower than the WT-catalytic domain whereas significant fluctuation was observed in the RMSD values of V2906A with several spikes of ~ 1 nm from 25 to 32 ns period. We have monitored the RMSF to calculate the average fluctuation of amino acid residue in order to determine the mutation’s effect on the protein residues dynamic behavior. From Fig. 6(B1–B3), it can be inferred that residue level fluctuations for V1736G, V2906A and V216G were quite high, up to ~ 0.5 nm, ~ 0.6 nm and ~ 1 nm respectively when compared with native proteins and other mutations. Analysis of the fluctuations also revealed that the greatest degree of flexibility was shown by the V2906A mutant. We have also analyzed the radius of gyration (R_g_) for the WT proteins along with its associated mutations contributing to their compactness shown in Fig. 6C1–C5. From Table 6, it can be concluded that V1687G and V216G had approximately similar compactness as of their respective WT proteins. Rather than these, all the mutations possessed higher Rg values than their WT proteins suggesting their structural destabilizing effects caused by the mutations, ultimately leading to the loss of protein compactness. Finally, the solvent-accessible surface areas (SASAs) were analyzed to understand the changes in the protein volume upon mutation. V1687G, I2865T and V2906A mutants showed increased SASA values compared to their WT proteins. The decreased SASA value in the remaining mutants denotes their relatively shrunken nature as compared to the WT structures. The change of SASA value of WT and mutant proteins with time is shown in Fig. 6D1–D3. Similar results were observed in case of the 2nd and 3rd MD runs with no significant deviation as seen from Table 7, Supplementary Figs. S3 and S4.

**Table 6:** The average values of RMSD, RMSF, Rg and SASA for WT and mutant proteins.

Protein/complex	MD runs	RMSD (nm)	RMSF (nm)	Rg (nm)	SASA (nm^2^)
WT-BRCT	1st run	~ 0.10	~ 0.34	~ 1.94	~ 116
	2nd run	~ 0.11	~ 0.35	~ 1.94	~ 121
	3rd run	~ 0.14	~ 0.38	~ 1.95	~ 118
V1687G	1st run	~ 0.11	~ 0.47	~ 1.94	~ 119
	2nd run	~ 0.13	~ 0.44	~ 1.94	~ 119
	3rd run	~ 0.14	~ 0.44	~ 1.94	~ 114
V1736G	1st run	~ 0.14	~ 0.45	~ 1.96	~ 114
	2nd run	~ 0.10	~ 0.40	~ 1.94	~ 118
	3rd run	~ 0.15	~ 0.41	~ 1.92	~ 113
WT-Catalytic domain	1st run	~ 0.45	~ 0.50	~ 1.96	~ 160
	2nd run	~ 0.35	~ 0.80	~ 1.99	~ 165
	3rd run	~ 0.32	~ 0.76	~ 1.98	~ 165
I2865T	1st run	~ 0.33	~ 0.40	~ 2.02	~ 164
	2nd run	~ 0.29	~ 0.45	~ 1.96	~ 165
	3rd run	~ 0.39	~ 0.46	~ 2.17	~ 169
V2906A	1st run	~ 0.45	~ 0.68	~ 2.07	~ 168
	2nd run	~ 0.29	~ 0.64	~ 1.99	~ 168
	3rd run	~ 0.31	~ 0.66	~ 1.95	~ 166
WT-DBD	1st run	~ 0.12	~ 0.43	~ 1.70	~ 115
	2nd run	~ 0.15	~ 0.57	~ 1.71	~ 116
	3rd run	~ 0.12	~ 0.55	~ 1.70	~ 112
V216G	1st run	~ 0.16	~ 0.62	~ 1.70	~ 112
	2nd run	~ 0.16	~ 0.63	~ 1.70	~ 114
	3rd run	~ 0.19	~ 0.53	~ 1.71	~ 116
L194H	1st run	~ 0.16	~ 0.51	~ 1.71	~ 114
	2nd run	~ 0.12	~ 0.57	~ 1.71	~ 116
	3rd run	~ 0.11	~ 0.57	~ 1.71	~ 116

**Table 7:** MM/PBSA calculations of binding free energy for six mutant-PK083 complexes from 1st MD production run.

Complexes	Van der Waal energy (KJ mol^−1^)	Electrostatic energy (KJ mol^−1^)	Polar solvation energy (KJ mol^−1^)	SASA energy (KJ mol^−1^)	Binding energy (KJ mol^−1^)
V1687G-PK083	− 162.942 ± 8.328	− 4.006 ± 4.198	72.501 ± 10.932	− 15.973 ± 0.812	− 110.419 ± 11.896
V1736G-PK083	− 176.793 ± 9.540	− 29.386 ± 4.914	111.041 ± 11.923	− 15.701 ± 0.803	− 110.839 ± 12.909
V216G-PK083	− 163.826 ± 9.659	− 14.571 ± 4.866	81.793 ± 11.348	− 16.607 ± 0.861	− 113.211 ± 10.362
L194H-PK083	− 168.406 ± 8.744	− 29.885 ± 8.523	121.305 ± 13.577	− 15.397 ± 0.843	− 92.383 ± 11.060
I2865T-PK083	− 128.608 ± 9.752	− 11.997 ± 5.278	55.265 ± 7.757	− 14.316 ± 1.079	− 99.656 ± 9.611
V2906A-PK083	− 86.402 ± 9.708	− 27.661 ± 11.869	80.415 ± 20.936	− 9.403 ± 1.008	− 43.051 ± 12.340

### Stability of the docked protein–ligand complexes

**Figure 7:**
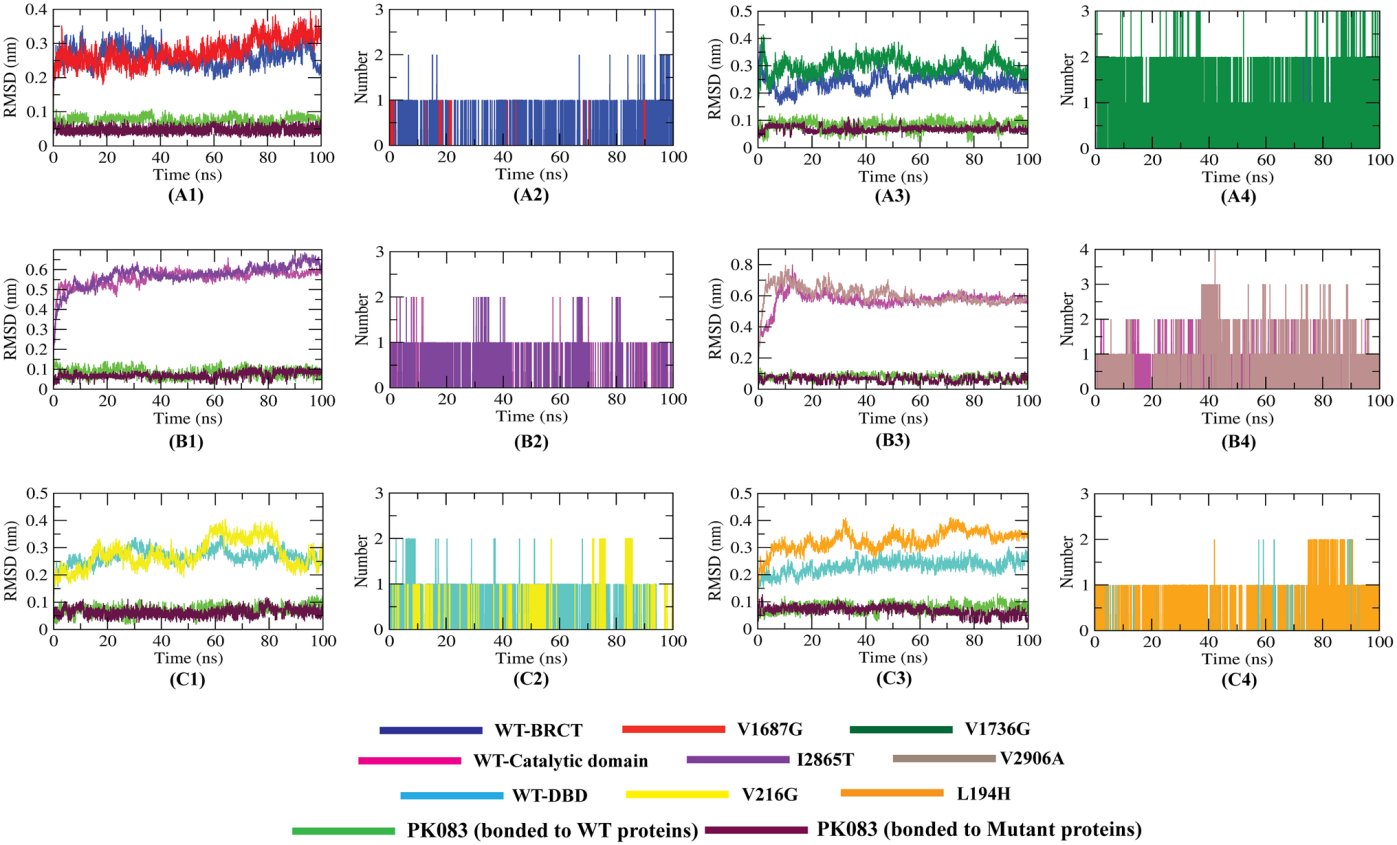
MD simulation results (1st independent run) of WT and mutant-PK083 complexes. (**A1,A3,B1,B3,C1,C3**) RMSD of PK083 and protein backbone of WT and mutant proteins obtained from 100 ns MD simulation. (**A2,A4,B2,B4,C2,C4**) Hydrogen bond analysis between PK083 with the WT and mutants.

The stability of the docked complexes during simulation period were assessed analyzing RMSD of the protein backbone and ligand structure as well as the hydrogen bonds analysis formed by PK083 with the WT and mutants (Fig. 7). We performed 3 independent production runs of the protein–ligand complexes, the 1st production run included both the WT and mutant-PK083 complexes (Fig. 7) while the 2nd and 3rd production run included the mutant-PK083 complexes (Supplementary Figs. S5, S6).

**Figure 8:**
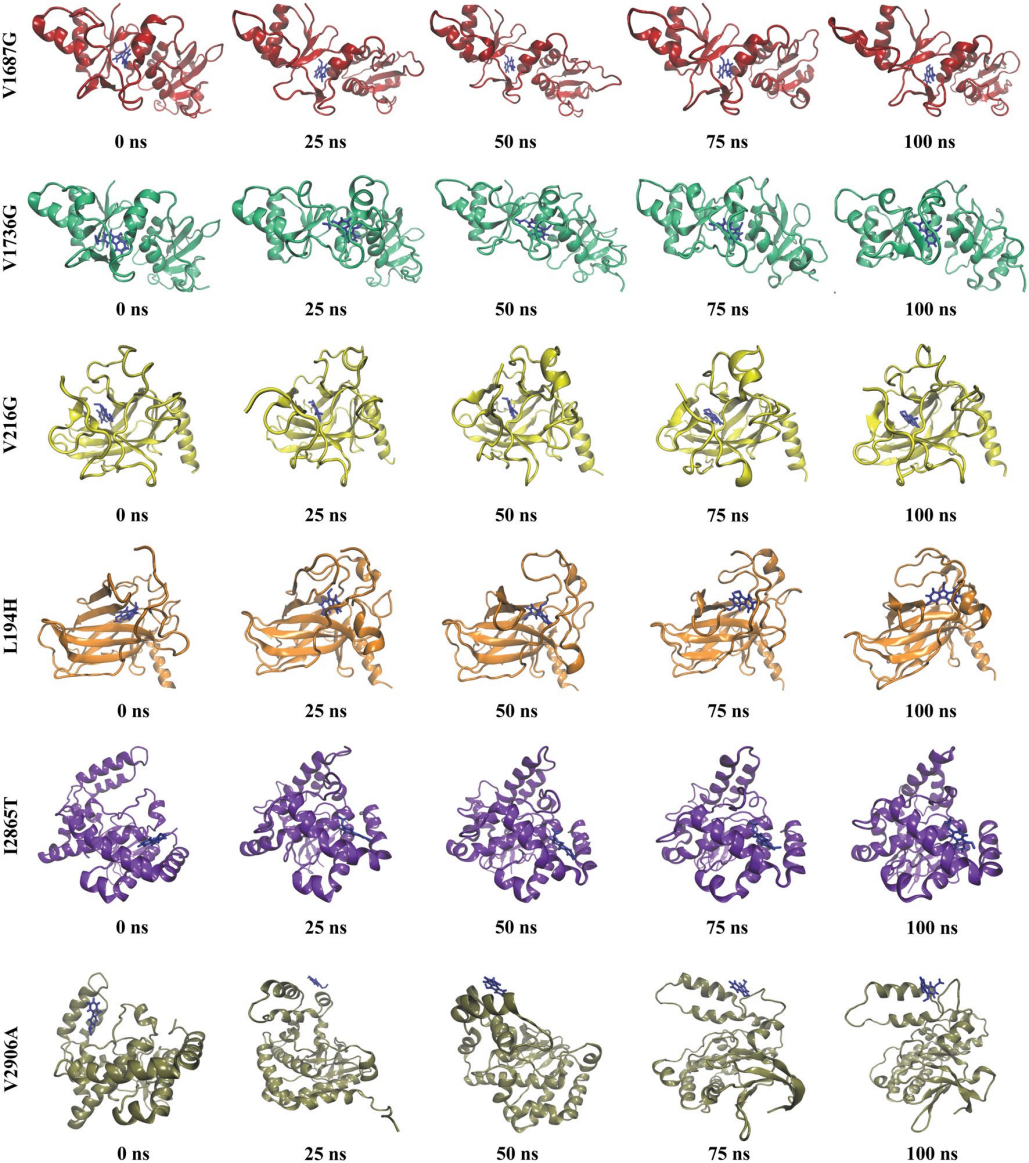
Binding pose of PK083 over the course of 100 ns simulation (1st MD production run) with the mutants.

It was observed that, all the WT and mutant proteins exhibited similar backbone RMSD except the L194H mutant, the WT (L194) protein were more stable compared to the mutant. In case of the six mutants, it was found that rather than the V2906A complex, all the remaining protein–ligand complexes were stable. For the five stable complexes, the ligand RMSD was less than 0.1 nm indicating the initial ligand-backbone contacts remained intact during the simulation period. In case of V2906A, the PK083 showed multiple binding orientations and it re-equilibrated several times during the 100 ns simulation (Fig. 7B3). It was also observed that the protein backbone RMSD of I2865T and have significantly decreased upon binding of PK083 than the apo form shown in Fig. 7B1. Further, we calculated the number of hydrogen bonds formed during the simulations period for the WT and mutant complexes, presented in Fig. 7A2,A4,B2,B4,C2,C4 as hydrogen bonding is one of the principal components responsible for the molecular interactions in biological systems. In the V1736G-PK083 complex, highest number of conformations formed up to three hydrogen bonds during the simulation. A very few conformations showed less than two hydrogen bonds. Except the V1687G-PK083 complex, the conformations of the rest of the complexes formed up to two hydrogen bonds throughout the simulation of all the 3 production runs. Moreover, the snapshot of conformers from the 1st MD run showed that PK083 remained in the binding sites of mutants throughout the entire simulation process (Fig. 8). We have also analyzed the interactions of PK083 with the mutants at the final timeframe (100 ns) and found that 2 interactions of V1687G, 3 interactions of L194H, 4 interactions of V1736G, I2865T and V216G with PK083 were stable at the end of the simulations. Although there were no interactions proposed in docking remain stable at the end of the simulation in case of V2906A mutant, it formed 6 interactions with PK083 near the mutant residue. The simulation trajectories of all the complexes were further exploited to study the interaction between the mutants and PK083.

### Post molecular dynamics binding free energy calculation

**Figure 9:**
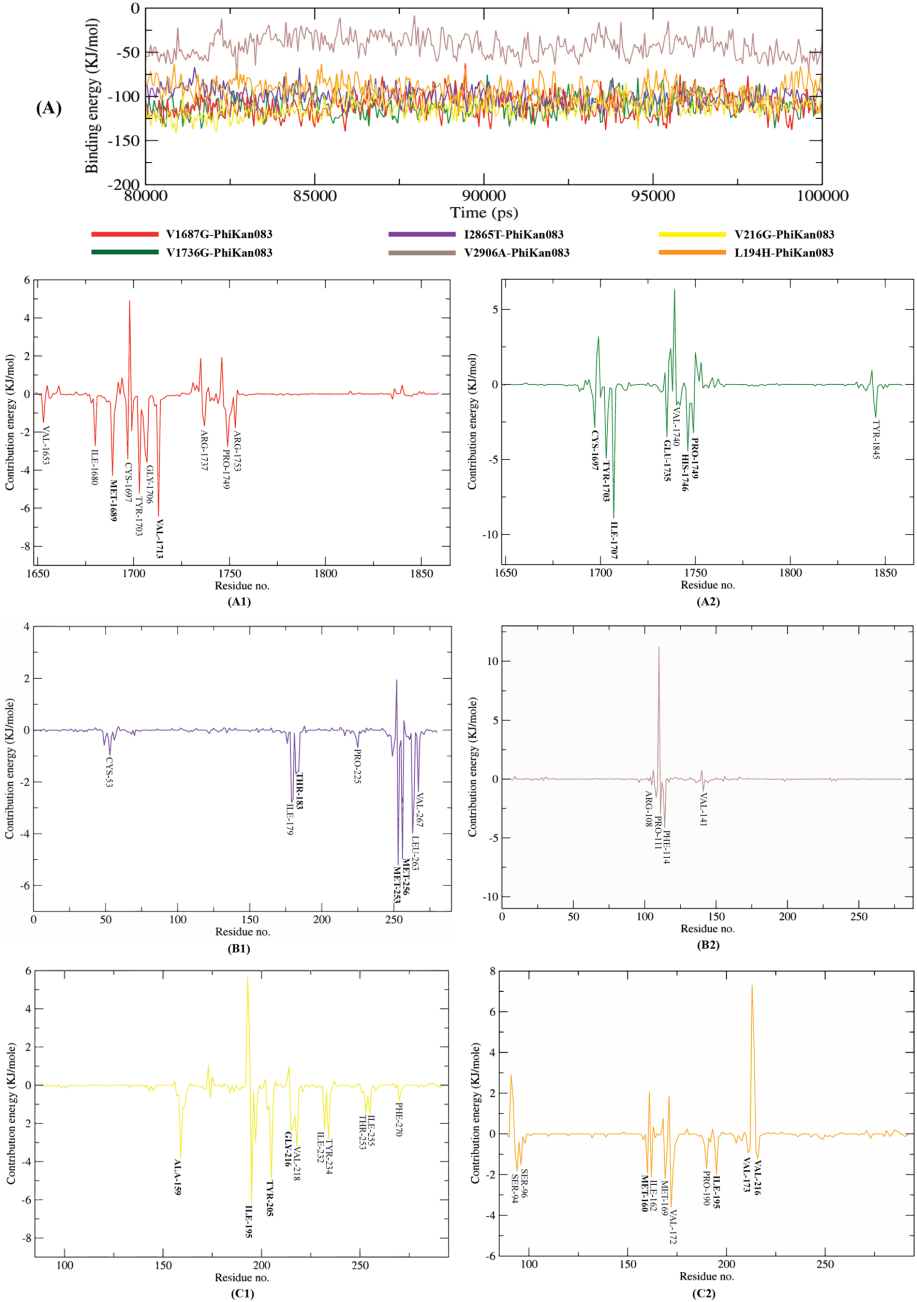
Graphical representation showing contribution energy plot of each residue of the six mutants, (A1) V1687G, (A2) V1736G, (B1) I183T (I2865T), (B2) V224A (V2906A), (C1) V216G and (C2) L194H. The residues that were involved in molecular docking are shown in bold.

We calculated the binding free energy of the last 20 ns of 1st MD production run of the mutant-PK083 complexes with an interval of 50 ps (picoseconds) from MD trajectories using MM/PBSA method. We also utilized the MmPbSaStat.py script included in g_mmpbsa package calculating the average free binding energy and its standard deviation/error from the simulation output files (Table 7). The interaction between a ligand and protein is shown in the form of binding energy where lesser the binding energy, the better is the binding of the ligand and protein. The cumulative sum of van der Wall, electrostatic, polar solvation, and SASA energy is the final binding energy. PK083 showed the least binding free energy (− 113.211 kJ/mol) with the V216G variant of p53 among the mutants. The carbazole derivative showed almost similar binding free energy with the two variants of BRCT. By plotting the binding energy versus time graphs, a comparison of the binding free energies of all the six complexes were made, shown in Fig. 9. These results verify that PK083 might possess stabilizing effect on majority of the deleterious mutations of BRCA1, ATM and TP53 effectively.

Further, we identified the contribution of each residue of the six mutants in terms of binding free energy to the interaction with PK083. By decomposing the total binding free energy of the system into per residue contribution energy, the contribution of each residue was calculated, shown in Fig. 9. This gives us an insight into the ‘hotspot’ residues that contributes favorably to the binding of this molecule to the mutants. It was found that except the V2906A variant of ATM, more than five residues of the remaining mutants contributed higher than − 1 kJ/mol binding energy. These identified key residues from our analysis will facilitate the study of mutation sites stabilization of three significant domains of these proteins.

## Discussion

In human genome, non-synonymous single nucleotide polymorphisms account for about 50% of allele variation of all hereditary diseases^44^. It can help in improving medication strategies by facilitating more tailored personalized treatment to patients^45^. Also, new compounds can be tested to correct the effects of those mutations studying the effects generated by nsSNPs in disease-associated proteins. Identification of such nsSNPs responsible for specific phenotypes using molecular approaches is time-consuming and expensive^46^. Bioinformatics predicting approaches can help in narrowing down the number of high-risk pathogenic nsSNPs to be screened in genetic association studies, and in a better understanding of the function and structure of protein products.

In this study, we performed an intensive in silico evaluation to identify pathogenic nsSNPs of BRCA1, ATM and TP53 genes using a wide variety of computational tools. We selected these genes based on gene enrichment analysis from a list of nine genes. Only two studies have been carried out to evaluate the nsSNPs of human BRCA1 and ATM gene previously. Previously, a mutation of proline to serin at position 1812 as a main target mutation in the BRCA1 gene was reported analyzing only 65 nsSNPs of this gene in 2007^47^. Also, upon analyzing the functional impact of 168 nsSNPs of ATM gene using two computational tools, SIFT and PolyPhen in 2012, six nsSNPs were classified as highly damaging substitutions^48^. We expanded our study to include all the nsSNPs currently available in dbSNP database and hypothesized that a more reliable and precise estimation of a substitution consequence could be provided by using a variety of computational methods based on different algorithms to filter the pathogenic and neutral variants.

In this study, we retrieved all the available nsSNPs of the corresponding three genes and annotated them using nine computational tools to distinguish between the functional and neutral variants. Assessing the pathogenicity of functional nsSNPs, we filtered those that occur in the conserved domains of respective proteins. Further, evolutionary conservation analysis revealed that majority of the pathogenic nsSNPs occupy conserved amino acid positions whether they decrease or increase protein stability. The nsSNPs greatly decreasing the stability of proteins were finally selected as the high-risk ones. Combining these results, we found that V1687G and V1736G variants of BRCA1, I2865T and V2906A variants of ATM, V216G and L194H variants of TP53 were highly damaging mutations that greatly decrease protein stability and might alter their respective protein functions (Table 2). All these six variants were found to occur in BRCT domain, catalytic domain and DNA-binding domain of BRCA1, ATM and p53 respectively (Fig. 3). The missense mutations in these domains might cause severe consequences disrupting their ability of functioning. The harmful polymorphic mutations are mainly found to be located in helixes and coil regions of a protein structure^49^. Our secondary structure analysis also revealed that three of these six mutations occur in coil regions, two in β-strand region and one in α-helix region which is in accordance with the previous recognition. Therefore, these mutations might result in significant distortion of the backbone over a turn leading to the likelihood of impaired molecular assembly.

Hence, these novel findings encouraged us to study how the dynamic properties differ from WT and mutant amino acids using MD simulation analysis. The RMSD and RMSF analysis in agreement to each other revealed that all the variants except the I2865T, decreased stability and increased the flexibility of protein (Fig. 6A1–B3). The radius of gyration revealed that the WT proteins showed higher level of compactness throughout the time, whereas all the variants showed differential level of compactness (Fig. 6C1–C3). The SASA analysis showed both increased and reduced volumes gained by the mutants and thereby might be responsible for change in function of protein (Fig. 6D1–D3). Based on the simulation study, we demonstrated that the variants V1687G, V1736G, V2906A, V216G and L194H imparted changes in the native conformation or structure of the BRCA1, ATM and TP53 proteins in any sense of behavior and hence speculated to affect the respective protein function and structure in damaging manner.

Further, we carried out our investigation to stabilize the high-risk mutations using a small molecule inhibitor PK083. Mutation site specific molecular docking analysis revealed that PK083 had a strong binding affinity towards all the six mutation sites of three proteins (Table 5, Fig. 5). More than five interactions were observed with PK083 in the mutation sites of all the mutant proteins. Highest docking energy of − 99.5 kcal/mol was observed with the WT-V2906 protein forming a carbon hydrogen bond with PK083. In case of the mutants, PK083 showed highest docking energy of − 72.3 kcal/mol forming a hydrogen bond with mutant residue. For the validation of docking process, we performed MD simulation (3 times) of six mutant-PK083 complexes over 100 ns and findings demonstrated that PK083 showed RMSD of less than 0.1 nm and no disassociation of bound PK083 is observed throughout the 100 ns simulations (Fig. 7A1,A3,B1,B3,C1,C3). Moreover, 2 interactions of V1687G, 6 interactions of V1736G, 3 interactions of I2865T, 4 interactions of both V216G and L194H with PK083 remained stable during the 100 ns MD simulation. Stability of PK083 during MD simulation were also supported by several H-bonds estimations (Fig. 7A2,A4,B2,B4,C2,C4). The results of MM/PBSA indicated that PK083 binds to all the six mutants efficiently as they exhibit good binding free energies (Table 7). We also identified several key residues essential for binding of PK083 to the mutation sites that will provide valuable insight in drug development against these deleterious mutations (Fig. 9).

Our further analysis from PolymiRTS database identified both the target sites disrupted and created by SNPs and INDELs in miRNA seeds. Four miRNAs, hsa-miR-6796-3p, hsa-miR-1233-5p, hsa-miR-525-3p and hsa-miR-548i were affected by the SNPs rs3745198, rs71309450, rs190453265, rs201549145 respectively having high conservation score (Table 3). As a result, the areas affected by those SNPs might have evolutionary important function.

Our study reports that six nsSNPs (V1687G and V1736G of BRCA1, I2865T and V2906A of ATM, V216G and L194H of TP53) in the BRCT, Catalytic and DNA-binding domains are highly damaging to the structure and function of three proteins. We also found that these mutants are drug targets for PK083 as revealed by molecular interaction results. Several miRNAs were affected by SNPs in the 3′ and 5′ untranslated regions of ATM and TP53 genes and hsa-miR-6796-3p, hsa-miR-1233-5p, hsa-miR-525-3p and hsa-miR-548i due to their higher likelihood of causing disruption or creation in the miRNA target site. Therefore, our findings could provide a cornerstone to the study of potential therapeutic inventions upon clinical-trial and experimental mutational studies.

## Methods

**Figure 10:**
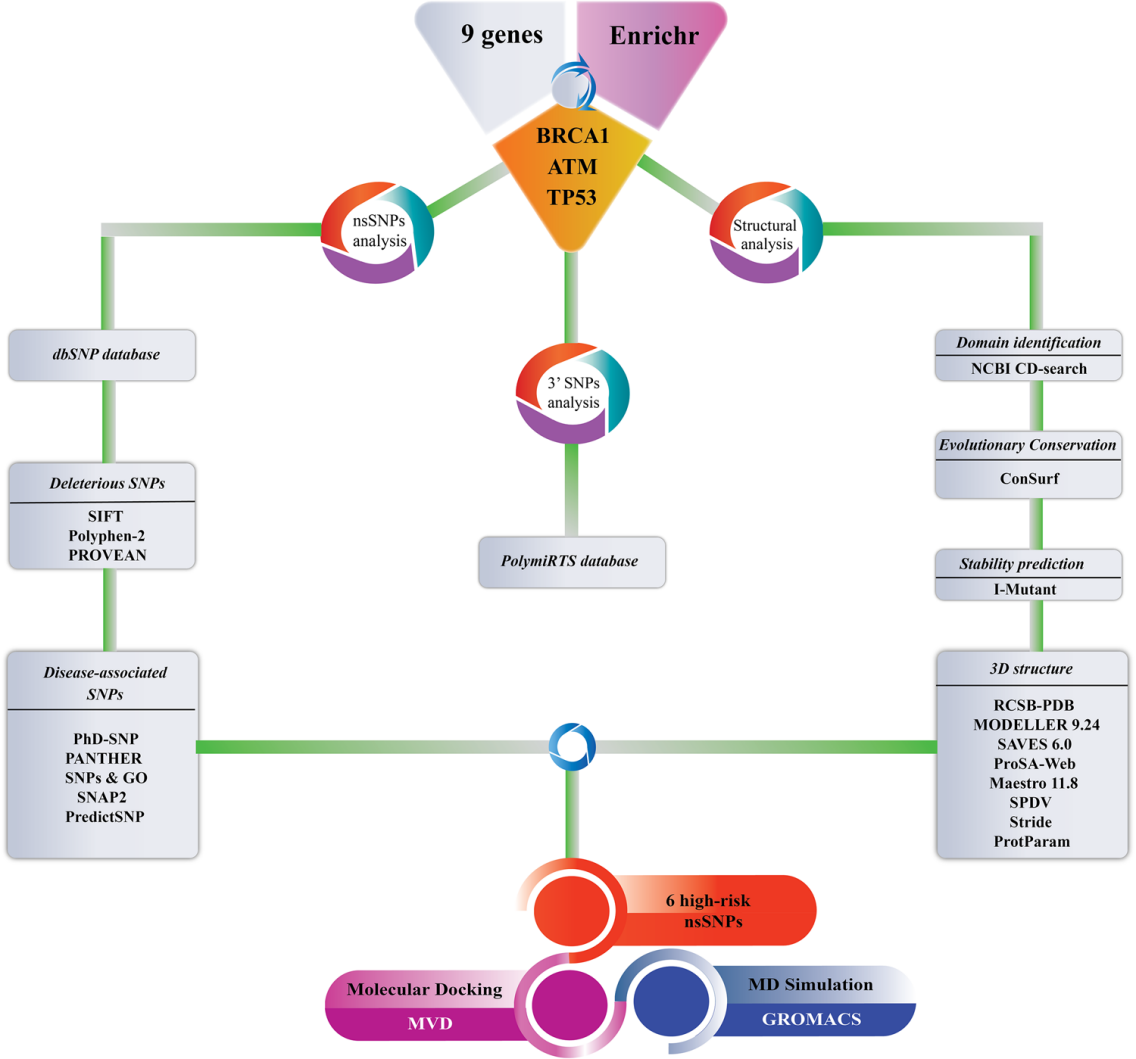
Schematic workflow of identifying the deleterious SNPs of cancer susceptibility genes.

A step-wise protocol was followed to identify the pathogenic nsSNPs of the selected cancer susceptibility genes. The work flow is depicted in Fig. 10.

### Gene enrichment analysis

Gene symbols of nine selected genes were uploaded to Enrichr^50^ web-server that evaluates the biological properties of genes based on enrichment analysis. The z-score method was used for computation of enrichment and a combined scoring method was used to compute a combined P value from Fisher’s exact test^50^.

### Retrieval of nsSNPs

Information of nsSNPs (SNP rs IDs, position, and residue changes) of the selected genes was retrieved from the NCBI dbSNP database^51^. The “Missense” filter was used in the function class and all the nsSNPs were retrieved for analysis.

### Prediction of deleterious nsSNPs

Three computational tools, SIFT^52^, PolyPhen-2^53^, and PROVEAN^54^ were used for the identification of deleterious nsSNPs. The SIFT (Sorting Intolerant from Tolerant) webserver predicts tolerated or deleterious substitution for every position of the query sequence based on multiple alignment information. PolyPhen-2 (Polymorphism Phenotyping-2) calculates the functional significance of an allele change by a set of supervised learning algorithms called the Naive Bayes classifier. PROVEAN (Protein Variation Effect Analyzer) also classifies the effect of an amino acid substitution, in-frame insertions and deletions on the biological function of a query protein.

### Prediction of disease-associated nsSNPs

Five different web tools, PhD-SNP^55^ (Predictor of human Deleterious Single Nucleotide Polymorphisms), PANTHER^56^ (Protein ANalysis THrough Evolutionary Relationships), SNPs&GO^57^, SNAP2^58^ (Screening for Nonacceptable Polymorphisms 2) and PredictSNP^59^ were used to detect the disease-associated nsSNPs from the selected genes. These tools use various algorithms such as support vector machines (PhD-SNP and SNPs&GO), Hidden Markov-Model based statistical modeling (PANTHER), and neural network (SNAP2) to predict the SNPs with functional effects upon utilizing the user-provided sequence information. The PredictSNP tool classifies nsSNPs based on consensus method that combines the output of six different prediction webtools (MAPP, nsSNP Analyzer, PANTHER, PhD-SNP, PolyPhen-1, PolyPhen-2, SIFT, and SNAP) to analyze the effect of nsSNPs on protein function.

### Identification of nsSNPs in conserved protein domains

Protein domains are distinct functional and structural units in a protein. They are responsible for a particular function or interaction which contributes to the overall role of a protein^24^. Protein domains can be highly altered by the presence of SNPs and proteins with these domain-altering SNPs contain highly connected nodes in various cellular pathways^60^. So, we intended to find out the nsSNPs that occur on the domains of the proteins encoded by the selected genes. Domain search was carried out at NCBI’s CD-Search tool (https://www.ncbi.nlm.nih.gov/Structure/cdd/wrpsb.cgi) to find out the conserved domains of the selected proteins^61^.

### Evolutionary conservation analysis

The evolutionary conservation of amino acid substitution was analyzed using ConSurf web server^62^. This server uses an empirical Bayesian inference to automatically analyze evolutionary conservation of amino acid substitutions in protein. The corresponding ConSurf conservation score ranges from 1 to 9, where 1 designates rapidly evolving (variable) regions, 5 designates mildly evolving regions, and 9 indicates conserved regions^63^. The exposed residues having high scores are thought to be functional residues, whereas the buried residues having high scores are considered structural.

### Prediction of changes in protein stability

I-Mutant 2.0 was used to analyze the protein stability changes upon nsSNPs. The tool is based on support vector machine (SVM) that provides a free energy change value (ΔΔG) of protein after and before mutation as output and uses data derived from ProTherm database which is one of the most comprehensive of experimental data on protein mutations^64,65^. ΔΔG value of less than ‘0’ indicates that the variant decreases the protein stability and ΔΔG value of greater than 0 indicates that the variant increases the protein stability.

### Functional effect analysis of SNPs in 3′ and 5′ untranslated regions (UTR)

PolymiRTS v3.0 database was used to characterize the SNPs in 3′ and 5′ regions and to analyze the functional impact of genetic polymorphisms in miRNA seed regions and miRNA target sites. This is an integrated platform for analyzing SNPs that affect miRNA. We entered the gene symbols of the selected genes and acquired a list of the miRNAs affected by these mutations that might lead to a decrease/increase of the expression of genes.

### Homology modeling and structural analysis of variants

To evaluate whether the high risk nsSNPs alter the WT structure of protein domains, we analyzed the three-dimensional (3D) structures of both WT and mutant domains. The PDB database (https://www.rcsb.org/) was used to retrieve the available protein structures whereas MODELLER 9.22^66^ was used to generate the 3D structures that were not available in the database. The DOPE and GA341 objective functions were used to choose the best structure from MODELLER, where higher GA341 and/or lower DOPE indicates higher quality of a generated model. The best modelled structures were refined through the GalaxyRefine^67^ server. The resultant structures were verified by PROCHECK^68^ and ERRAT^69^ tool from SAVES 6.0 server and ProSA-web^70^ analysis program. Later, the Maestro 11.8 tool of Schrödinger suite^71^ was used to compare the WT protein structures with the mutants computing the root mean square deviation (RMSD). Higher RMSD indicates greater variation between WT and mutant structures^72^. All the structures were visualized by Pymol and Maestro 11.8. Further, the Stride^73^ web-server was employed to view the mutation location in secondary structures while the ProtParam^74^ server was used for the analysis physicochemical properties of WT and mutant proteins.

### Molecular docking analysis

We used Molegro Virtual Docker^75^ (MVD) for molecular docking study to view the binding affinity of a small molecule stabilizer with the mutant domains. The software is unified with high potential Piece Wise Linear Potential (PLP) and MVD scoring function. A carbazole derivative, PK083 (1-(9-ethylcarbazol-3-yl)-*N*-methylmethanamine) was used as ligand. Carbazole based small-molecules were tested to act as stabilizers in restoring the function of several mutants of p53 DBD^42,43^. So, we tested the stabilizing capability of PK083 to the highly damaging mutants of our study. All the mutant proteins were subjected to energy minimization using SwissPdb viewer^76^ and the ligand structure was optimized with MMFF94 force field using steepest descent algorithm prior to docking. The binding site was defined covering the mutant residue to assess the binding affinity of PK083 to each mutation region. The docking processes were composed of maximum iteration of 1500, maximum population size of 50 and Grid solution of 0.3. Further, we carried out post docking processes by hydrogen bonds optimization and energy minimization, simplex evolution at max steps 300 and neighbor distance technical setting fast at 1.00. The energy of the receptor-ligand complexes was minimized using Nelder Mead Simplex Minimization. Later on, the interactions of mutants with ligand were visualized in Discovery Studio 4.1.

### Molecular dynamics (MD) simulations and MM/PBSA analysis

Molecular dynamics simulation of the WT and mutant protein structures was performed using GROMACS 5.1.4 version^77^ and Linux 5.4 package. The GROMOS96 54a7^78^ forcefield was selected as the force field for proteins and the ligand topologies were generated from the Automated Topology Builder version 3.0^79^ (ATB) server. Due to the enhanced capacity of the backbone NH and CO groups to form hydrogen bonds with each other in the GROMOS96 54A7 parameter set, this force field reproduces the folding equilibria slightly better and can sample more 314-helical or hairpin conformations than the previous 53A6 or 45A3 force fields^80^. Also, based on fitting to a large set of high-resolution crystal structures, the torsional angle terms were reparametrized in this parameter set^81^. The proteins and mutants-ligand complexes were solvated using simple point charge (SPC) water molecules in a rectangular box where every structure was placed in the center at least 1.0 nm from the box edges. Required number of Na+ and Cl− ions were added to make the simulation system electrically neutral. The salt concentrations were set to 0.15 mol/L in all the systems. The solvated systems were subjected to energy minimization for 5000 steps using the steepest descent method. Afterwards, three steps were conducted in the MD simulation: NVT (constant number of particles, volume, and temperature) series, NPT (constant number of particles, pressure, and temperature) series, and the production run. The NVT and the NPT series were conducted at a 300 K temperature and 1 atm pressure for the duration of 100 ps. V‐rescale and Parrinello‐Rahman were selected as the thermostat and barostat respectively of the performed simulation. Finally, 3 independent production runs of nine proteins (WT and mutants) and six protein–ligand complexes were performed at 300 K for a duration of 100 ns (nanoseconds) in a supercomputing system provided by the Bioinformatics Division of National Institute of Biotechnology (NIB), Bangladesh. Thereafter, a comparative analysis was performed between WT and mutants measuring root mean square deviation (RMSD), root mean square fluctuation (RMSF), radius of gyration (Rg), solvent accessible surface area (SASA) and hydrogen bonds. Qtgrace program was used to represent all these analyses in the form of plots^82^. Further, the g_mmpbsa^83^ package of GROMACS was used to calculate the MM/PBSA (Molecular Mechanics/Poisson Boltzmann Surface Area) binding free energies followed by final MD run to get a more detailed overview of the biomolecular interactions between the mutated proteins and ligand. The tool was tested on 37 structurally divergent HIV-1 protease inhibitor complexes by performing comparison of the calculated relative binding energy with the experimental binding free energies^83^. Also, the results obtained using g_mmpbsa package were comparable to results obtained with the AMBER package in general within differences of 1–2 kcal/mol. Furthermore, the package can be used to approximate the energy contribution per residue to the binding energy and it has been used to identify the crucial residues for binding a range of inhibitors with HIV-1 protease^83^. The free solvation energy (polar and nonpolar solvation energies) and potential energy (electrostatic and Van der Waals interactions) of each protein–ligand complex were analyzed to determine the total ΔG_bind_. The binding energies were calculated using the following equation in this method:

$$ \Delta {\text{G}}_{{{\text{binding}}}} = {\text{ G}}_{{{\text{complex}}}} - \, \left( {{\text{G}}_{{{\text{protein}}}} + {\text{ G}}_{{{\text{ligand}}}} } \right). $$

Here, the ΔG_binding_ = the total binding energy of the protein–ligand complex, G_protein_ = the binding energy of free protein, and G_ligand_ = the binding energy of unbounded ligand.

## Conclusion

BRCA1, ATM and TP53 protein plays an important role as tumor suppressor in several cancer types. The structural conformation of the functional domains is very crucial for exerting their functional role. This in silico study of the functional SNPs of BRCA1, ATM and TP53 provides significant insight into the damaging effects that the nsSNPs might cause to these proteins. Our study reports that six nsSNPs (V1687G and V1736G of BRCA1, I2865T and V2906A of ATM, V216G and L194H of TP53) in the BRCT, Catalytic and DNA-binding domains are highly damaging to the structure and function of three proteins. We also found that these mutants are drug targets for PhiKan083 as molecular interaction results were evaluated by MD simulation and MMPBSA study. miRNAs were affected by SNPs in the 3′ and 5′ untranslated regions of ATM and TP53 genes and four are noteworthy, hsa-miR-6796-3p, hsa-miR-1233-5p, hsa-miR-525-3p and hsa-miR-548i due their high conservation score. This is the first in silico analysis that combinedly analyzes the impacts of nsSNPs on the structure and function of three cancer susceptibility proteins and predicts stabilizing possibility. Our findings will guide in the study of potential therapeutic inventions upon clinical-trial and experimental mutational studies.

## Supplementary Information

Supplementary Information.

## Data Availability

All data generated and analyzed during this study are included in this article.

## References

[1] Calderón-Garcidueñas AL, Ruiz-Flores P, Cerda-Flores RM, Barrera-Saldaña HA (2005). Clinical follow up of Mexican women with early onset of breast cancer and mutations in the BRCA1 and BRCA2 genes. Salud Publica Mex..

[2] Ahmed M, Rahman N (2006). ATM and breast cancer susceptibility. Oncogene.

[3] Olivier M, Hollstein M, Hainaut P (2010). TP53 mutations in human cancers: Origins, consequences, and clinical use. Cold Spring Harbor Perspect. Biol..

[4] Bonadona V (2011). Cancer risks associated with germline mutations in MLH1, MSH2, and MSH6 genes in lynch syndrome. J. Am. Med. Assoc..

[5] Hendriks YMC (2004). Cancer risk in hereditary nonpolyposis colorectal cancer due to MSH6 mutations: Impact on counseling and surveillance. Gastroenterology.

[6] Foulkes WD, Flanders TY, Pollock PM, Hayward NK (1997). The CDKN2A (p16) gene and human cancer. Mol. Med..

[7] Hofstatter EW (2011). PALB2 mutations in familial breast and pancreatic cancer. Fam. Cancer.

[8] Martin SE (2009). BRCA1 E1644X: A deleterious mutation in an African American individual with early onset breast cancer. Breast Cancer Res. Treat..

[9] Futreal PA (1994). BRCA1 mutations in primary breast and ovarian carcinomas. Science.

[10] Malone KE (2006). Prevalence and predictors of BRCA1 and BRCA2 mutations in a population-based study of breast cancer in White and Black American women ages 35 to 64 years. Cancer Res..

[11] Rozman V, Kunej T (2018). Harnessing omics big data in nine vertebrate species by genome-wide prioritization of sequence variants with the highest predicted deleterious effect on protein function. Omi. A J. Integr. Biol..

[12] Krawczak M (2000). Human gene mutation database—A biomedical information and research resource. Hum. Mutat..

[13] Collins FS, Brooks LD, Chakravarti A (1998). A DNA polymorphism discovery resource for research on human genetic variation. Genome Res..

[14] Ng PC, Henikoff S (2002). Accounting for human polymorphisms predicted to affect protein function. Genome Res..

[15] Ronaghi M, Langaee T (2005). Single nucleotide polymorphisms: Discovery, detection and analysis. Per. Med..

[16] Kwok P-Y (2002). Single Nucleotide Polymorphisms.

[17] Singh R, Bhardwaj VK, Sharma J, Das P, Purohit R (2021). Identification of selective cyclin-dependent kinase 2 inhibitor from the library of pyrrolone-fused benzosuberene compounds: An in silico exploration. J. Biomol. Struct. Dyn..

[18] Bhardwaj VK, Singh R, Sharma J, Das P, Purohit R (2020). Structural based study to identify new potential inhibitors for dual specificity tyrosine-phosphorylation-regulated kinase. Comput. Methods Progr. Biomed..

[19] Bhardwaj VK, Purohit R, Kumar S (2021). Himalayan bioactive molecules as potential entry inhibitors for the human immunodeficiency virus. Food Chem..

[20] Singh R, Bhardwaj VK, Sharma J, Das P, Purohit R (2021). Discovery and in silico evaluation of aminoarylbenzosuberene molecules as novel checkpoint kinase 1 inhibitor determinants. Genomics.

[21] Bhardwaj V, Purohit R (2019). Computational investigation on effect of mutations in PCNA resulting in structural perturbations and inhibition of mismatch repair pathway. J. Biomol. Struct. Dyn..

[22] Bhardwaj VK, Purohit R (2020). A new insight into protein-protein interactions and the effect of conformational alterations in PCNA. Int. J. Biol. Macromol..

[23] Capriotti E, Altman RB (2011). Improving the prediction of disease-related variants using protein three-dimensional structure. BMC Bioinform..

[24] Basu MK, Poliakov E, Rogozin IB (2009). Domain mobility in proteins: Functional and evolutionary implications. Brief. Bioinform..

[25] Alshatwi AA, Hasan TN, Syed NA, Shafi G, Grace BL (2012). Identification of functional SNPs in BARD1 gene and in silico analysis of damaging SNPs: Based on data procured from dbSNP database. PLoS ONE.

[26] Chandrasekaran G (2017). In silico analysis of the deleterious nsSNPs (missense) in the homeobox domain of human HOXB13 gene responsible for hereditary prostate cancer. Chem. Biol. Drug Des..

[27] Hossain MS, Roy AS, Islam MS (2020). In silico analysis predicting effects of deleterious SNPs of human RASSF5 gene on its structure and functions. Sci. Rep..

[28] Deng N, Zhou H, Fan H, Yuan Y (2017). Single nucleotide polymorphisms and cancer susceptibility. Oncotarget.

[29] Miki Y (1994). A strong candidate for the breast and ovarian cancer susceptibility gene BRCA1. Science.

[30] Nissenkorn A, Ben-Zeev B, Islam MP, Roach ES (2015). Ataxia telangiectasia. Handbook of Clinical Neurology.

[31] Lavin MF (2004). Functional consequences of sequence alterations in the ATM gene. DNA Repair.

[32] Seidel JJ, Anderson CM, Blackburn EH (2008). A novel Tel1/ATM N-terminal Motif, TAN, is essential for telomere length maintenance and a DNA damage response. Mol. Cell. Biol..

[33] Tokino T (2004). Dual role of p53 in DNA binding. Cancer Biol. Ther..

[34] Joerger AC, Fersht AR (2010). The tumor suppressor p53: From structures to drug discovery. Cold Spring Harb. Perspect. Biol..

[35] Raj N, Attardi LD (2017). The transactivation domains of the p53 protein. Cold Spring Harb. Perspect. Med..

[36] Miller MP, Kumar S (2001). Understanding human disease mutations through the use of interspecific genetic variation. Hum. Mol. Genet..

[37] Doniger SW (2008). A catalog of neutral and deleterious polymorphism in yeast. PLoS Genet..

[38] Du K, Sharma M, Lukacs GL (2005). The ΔF508 cystic fibrosis mutation impairs domain-domain interactions and arrests post-translational folding of CFTR. Nat. Struct. Mol. Biol..

[39] Mayer S, Rüdiger S, Ang HC, Joerger AC, Fersht AR (2007). Correlation of levels of folded recombinant p53 in *Escherichia coli* with thermodynamic stability in vitro. J. Mol. Biol..

[40] Singh SM, Kongari N, Cabello-Villegas J, Mallela KMG (2010). Missense mutations in dystrophin that trigger muscular dystrophy decrease protein stability and lead to cross-β aggregates. Proc. Natl. Acad. Sci. U.S.A..

[41] Baretic D (2017). Structures of closed and open conformations of dimeric human ATM. Sci. Adv..

[42] Bauer MR (2020). Targeting cavity-creating p53 cancer mutations with small-molecule stabilizers: The Y220X paradigm. ACS Chem. Biol..

[43] Raghavan V, Agrahari M, Gowda DK (2019). Virtual screening of p53 mutants reveals Y220S as an additional rescue drug target for PhiKan083 with higher binding characteristics. Comput. Biol. Chem..

[44] Hamosh A, Scott AF, Amberger JS, Bocchini CA, McKusick VA (2005). Online mendelian inheritance in man (OMIM), a knowledgebase of human genes and genetic disorders. Nucleic Acids Res..

[45] Venkata Subbiah H, Ramesh Babu P, Subbiah U (2020). In silico analysis of non-synonymous single nucleotide polymorphisms of human DEFB1 gene. Egypt. J. Med. Hum. Genet..

[46] Chen X, Sullivan PF (2003). Single nucleotide polymorphism genotyping: Biochemistry, protocol, cost and throughput. Pharmacogenomics J..

[47] Rajasekaran R, Sudandiradoss C, Doss CGP, Sethumadhavan R (2007). Identification and in silico analysis of functional SNPs of the BRCA1 gene. Genomics.

[48] Doss CGP, Rajith B (2012). Computational refinement of functional single nucleotide polymorphisms associated with ATM gene. PLoS ONE.

[49] Kucukkal TG, Petukh M, Li L, Alexov E (2015). Structural and physico-chemical effects of disease and non-disease nsSNPs on proteins. Curr. Opin. Struct. Biol..

[50] Chen EY (2013). Enrichr: Interactive and collaborative HTML5 gene list enrichment analysis tool. BMC Bioinform..

[51] Bhagwat M (2010). Searching NCBI’s dbSNP database. Curr. Protoc. Bioinform..

[52] Ng PC, Henikoff S (2003). SIFT: Predicting amino acid changes that affect protein function. Nucleic Acids Res..

[53] Adzhubei I, Jordan DM, Sunyaev SR (2013). Predicting functional effect of human missense mutations using PolyPhen-2. Curr. Protoc. Hum. Genet..

[54] Choi Y, Chan AP (2015). PROVEAN web server: A tool to predict the functional effect of amino acid substitutions and indels. Bioinformatics.

[55] Capriotti E, Calabrese R, Casadio R (2006). Predicting the insurgence of human genetic diseases associated to single point protein mutations with support vector machines and evolutionary information. Bioinformatics.

[56] Thomas PD (2006). Applications for protein sequence-function evolution data: mRNA/protein expression analysis and coding SNP scoring tools. Nucleic Acids Res..

[57] Calabrese R, Capriotti E, Fariselli P, Martelli PL, Casadio R (2009). Functional annotations improve the predictive score of human disease-related mutations in proteins. Hum. Mutat..

[58] Bromberg Y, Rost B (2007). SNAP: Predict effect of non-synonymous polymorphisms on function. Nucleic Acids Res..

[59] Bendl J (2014). PredictSNP: Robust and accurate consensus classifier for prediction of disease-related mutations. PLoS Comput. Biol..

[60] Liu Y, Tozeren A (2010). Domain altering SNPs in the human proteome and their impact on signaling pathways. PLoS ONE.

[61] Yang M, Derbyshire MK, Yamashita RA, Marchler-Bauer A (2020). NCBI’s conserved domain database and tools for protein domain analysis. Curr. Protoc. Bioinform..

[62] Ashkenazy H (2016). ConSurf 2016: An improved methodology to estimate and visualize evolutionary conservation in macromolecules. Nucleic Acids Res..

[63] Zhang M, Huang C, Wang Z, Lv H, Li X (2020). In silico analysis of non-synonymous single nucleotide polymorphisms (nsSNPs) in the human GJA3 gene associated with congenital cataract. BMC Mol. Cell Biol..

[64] Bava KA, Gromiha MM, Uedaira H, Kitajima K, Sarai A (2004). ProTherm, version 4.0: Thermodynamic database for proteins and mutants. Nucleic Acids Res..

[65] Capriotti E, Fariselli P, Casadio R (2005). I-Mutant2.0: Predicting stability changes upon mutation from the protein sequence or structure. Nucleic Acids Res..

[66] Eswar N (2006). Comparative protein structure modeling using modeller. Curr. Protoc. Bioinform..

[67] Heo L, Park H, Seok C (2013). GalaxyRefine: Protein structure refinement driven by side-chain repacking. Nucleic Acids Res..

[68] Laskowski RA, MacArthur MW, Moss DS, Thornton JM (1993). PROCHECK: A program to check the stereochemical quality of protein structures. J. Appl. Crystallogr..

[69] Colovos C, Yeates TO (1993). Verification of protein structures: Patterns of nonbonded atomic interactions. Protein Sci..

[70] Wiederstein M, Sippl MJ (2007). ProSA-web: Interactive web service for the recognition of errors in three-dimensional structures of proteins. Nucleic Acids Res..

[71] Dagan-Wiener A (2017). Bitter or not? BitterPredict, a tool for predicting taste from chemical structure. Sci. Rep..

[72] Kuzmanic A, Zagrovic B (2010). Determination of ensemble-average pairwise root mean-square deviation from experimental B-factors. Biophys. J..

[73] Frishman D, Argos P (1995). Knowledge-based protein secondary structure assignment. Proteins Struct. Funct. Bioinform..

[74] Gasteiger E, Walke JM (2005). Protein identification and analysis tools on the ExPASy server. The Proteomics Protocols Handbook.

[75] Bitencourt-Ferreira G, de Azevedo WF, Clifton NJ (2019). Molegro virtual docker for docking. Methods in Molecular Biology.

[76] Guex N, Peitsch MC (1997). SWISS-MODEL and the Swiss-PdbViewer: An environment for comparative protein modeling. Electrophoresis.

[77] Abraham MJ (2015). Gromacs: High performance molecular simulations through multi-level parallelism from laptops to supercomputers. SoftwareX.

[78] Wei G, Baker N, Cui Q, Meuwly M, Ren P (2016). Differential geometry-based solvation and electrolyte transport models for biomolecular modeling: A review. Many-Body Effects and Electrostatics in Biomolecules.

[79] Stroet M (2018). Automated topology builder version 3.0: Prediction of solvation free enthalpies in water and hexane. J. Chem. Theory Comput..

[80] Schmid N (2011). Definition and testing of the GROMOS force-field versions 54A7 and 54B7. Eur. Biophys. J..

[81] Huang W, Lin Z, Van Gunsteren WF (2011). Validation of the GROMOS 54A7 force field with respect to β-peptide folding. J. Chem. Theory Comput..

[82] Mohammad T (2020). Virtual screening approach to identify high-affinity inhibitors of serum and glucocorticoid-regulated kinase 1 among bioactive natural products: Combined molecular docking and simulation studies. Molecules.

[83] Kumari R, Kumar R, Lynn A (2014). G-mmpbsa—A GROMACS tool for high-throughput MM-PBSA calculations. J. Chem. Inf. Model..

