# Supplementary material for: Pathogenic genetic variants from highly connected cancer susceptibility genes confer the loss of structural stability
Source: Sci Rep. 2021 Sep 28;11:19264. doi: 10.1038/s41598-021-98547-y (PMC8479081; doi:10.1038/s41598-021-98547-y)
Supplement: Supplementary file 1 — Supplementary Information. [file 41598_2021_98547_MOESM1_ESM.zip › Supplementary files-5-8-21/Supplementary file 10.pdf]

ConSeq Results

```
1           11          21          31          41
MDLSALRVEE  VQNVINAMQK  ILECPICLEL  IKEPVSTKCD  HIFCKFCMLK
eeeeeeebee  beebbebbee  bbebebbbeb  beeebeeebe  ebbbebbbbe
ff       f  sf s   sff   sf fs sf   sfffsff sf  fs     s f

51          61          71          81          91
LLNQKKGPSQ  CPLCKNDITK  RSLQESTRFS  QLVEELLKII  CAFQLDTGLE
bbeeeeeeee  beebbeeebee eeeeebeebe  ebbeebbebb  ebbebeebbe
  ff  f ff  sffsff  f   fffffsffsf  fssffs       ssf ff  f

101         111         121         131         141
YANSYNFAKK  ENNSPEHLKD  EVSIIQSMGY  RNRAKRLLQS  EPENPSLQET
bbeeeebeee  eeeeeeebee  ebebbbbbeb  eeebeebeee  eeeeeebeee
        f      f f        f s s f   fff                f

151         161         171         181         191
SLSVQLSNLG  TVRTLRTKQR  IQPQKTSVYI  ELGSDSSEDT  VNKATYCSVG
ebebebeebe  bbebbeeeee  eeeeeeebbb  ebbeeeeeee  beeeeebebe
                 ff          f  ss     fffff

201         211         221         231         241
DQELLQITPQ  GTRDEISLDS  AKKAACEFSE  TDVTNTEHHQ  PSNNDLNTTE
eeebbebeee  eeeeeeeeee  beebebebee  eebeebeeee  eeeeebeebe
                                f

251         261         271         281         291
KRAAERHPEK  YQGSSVSNLH  VEPCGTNTHA  SSLQHENSSL  LLTKDRMNVE
eebeeeeeee  eeebebbebe  beebeeeeeb  ebbeeeebeb  bbbeeeeebe
      f         f        ff  f   s  fs      f            f

301         311         321         331         341
KAEFCNKSKQ  PGLARSQHNR  WAGSKETCND  RRTPSTEKKV  DLNADPLCER
ebebeeeeee  eeeeeeeeee  eeeeeeebee  eeeeeeeeeb  eeebeeeeee
       f f       ff  f        f

351         361         371         381         391
KEWNKQKLPC  SENPRDTEDV  PWITLNSSIQ  KVNEWFSRSD  ELLGSDDSHD
eeeeeeeeee  eeeeeeeeeb  ebbbbeebbe  ebeebbeebe  ebbbeeeeee
                             ffss   fsff  f          f

401         411         421         431         441
GESESNAKVA  DVLDVLNEVD  EYSGSSEKID  LLASDPHEAL  ICKSERVHSK
eeeeeebebb  ebbebeeebe  eeeeeeeebe  bbeebeeebb  bbeeeeeeee
                             f f

451         461         471         481         491
SVESNIEDKI  FGKTYRKKAS  LPNLSHVTEN  LIIGAFVTEP  QIIQERPLTN
eeeeebeeeb  beeeeeeeee  eeebeebbee  bbbebbeeee  ebeeeeebee
       ffs  sffff ff

501         511         521         531         541
KLKRKRRPTS  GLHPEDFIKK  ADLAVQKTPE  MINQGTNQTE  QNGQVMNITN
ebeeeeeeee  ebeeeebbee  beeeeeeeee  eeeeeeeeee  eeeeebebee
  f ff       s fffssf    f
```

```
551        561        571        581        591
SGHENKTKGD SIQNEKNPNP IESLEKESAF KTKAEPISSS ISNMELELNI
eeeeeeeeee ebeeeeeeee eeeeeeeeeb eeebeebeee beebebebee
                        f

601        611        621        631        641
HNSKAPKKNR LRRKSSTRHI HALELVVSRN LSPPNCTELQ IDSCSSSEEI
eeeeeeeeee eeeeeeeeeb bbbebbbeee eeeeeeeebe beebeeeeeb
  f      f      f        f         f     f f s f  f ff

651        661        671        681        691
KKKKYNQMPV RHSRNLQLME GKEPATGAKK SNKPNEQTSK RHDSDTFPEL
eeeeeeeeee eeeeebebee eeeeeeeeee eeeeeeebee eebeeebeeb

701        711        721        731        741
KLTNAPGSFT KCSNTSELKE FVNPSLPREE KEEKLETVKV SNNAEDPKDL
ebeebeebbb eeeeeeeeee eeeeeeeeee eeeeeeebeb eeeeeeeeeb

751        761        771        781        791
MLSGERVLQT ERSVESSSIS LVPGTDYGTQ ESISLLEVST LGKAKTEPNK
bbeeeeeeee eeeeeeebbb bbeeeeeeee ebbbbbebee eeebeeeeee
             f            ff   f  s s

801        811        821        831        841
CVSQCAAFEN PKGLIHGCSK DNRNDTEGFK YPLGHEVNHS RETSIEMEES
ebeebebeee eeeebeeeee eeeeeeeebe eeeeeeeeeb eebbeebeee
                                                 f f f

851        861        871        881        891
ELDAQYLQNT FKVSKRQSFA PFSNPGNAEE ECATFSAHSG SLKKQSPKVT
eeeeebbeeb beeeeeebbb bbeeeeeeee ebbebbeebe ebeeeeeebe
f fff s f     fff ss  s f                s

901        911        921        931        941
FECEQKEENQ GKNESNIKPV QTVNITAGFP VVGQKDKPVD NAKCSIKGGS
eeeeeeeeee eeeeeeeeeb ebeebebebe ebeeeeeeee eeebeeeebb

951        961        971        981        991
RFCLSSQFRG NETGLITPNK HGLLQNPYRI PPLFPIKSFV KTKCKKNLLE
ebbeeeeeee eeeebbbeee eebeeeeeee eeebebeeeb eeebeeeeee

1001       1011       1021       1031       1041
ENFEEHSMSP EREMGNENIP STVSTISRNN IRENVFKEAS SSNINEVGSS
eebeeeeeee eeeeeeeebe bebeebeeee beeebbeeee eebbeeeeee
                                            f   f

1051       1061       1071       1081       1091
TNEVGSSINE IGSSDENIQA ELGRNRGPKL NAMLRLGVLQ PEVYKQSLPG
eeeeeeebee ebeeeeebee eeeeeeeeeb ebbbebebee eeebeeeeee
      f ff f ff f                         f f

1101       1111       1121       1131       1141
SNCKHPEIKK QEYEEVVQTV NTDFSPYLIS DNLEQPMGSS HASQVCSETP
```

```
eebeeeeeee eeeeebbebb eeebbebbbe eebeeeeeee ebbebbeeee
                                    f          f  ffff
1151       1161       1171       1181       1191
DDLLDDGEIK EDTSFAENDI KESSAVFSKS VQKGELSRSP SPFTHTHLAQ
eebeeeeebe eeeeebebeb eeebebbeee beeeeeeeee eebebbebee
  s                    f sf s f          f  f
1201       1211       1221       1231       1241
GYRRGAKKLE SSEENLSSED EELPCFQHLL FGKVNNIPSQ STRHSTVATE
eeeeebeeee eeeeeeeeee eebebbeebb beebeeeeee eeeeeebbee
       fff ffff  fff  f sf  f
1251       1261       1271       1281       1291
CLSKNTEENL LSLKNSLNDC SNQVILAKAS QEHHLSEETK CSASLFSSQC
ebeeeeeeeb eebeeebeee eeebbbbeee eeeebeeebe bbeebbebeb
                               f  f    f     s f  fsf
1301       1311       1321       1331       1341
SELEDLTANT NTQDPFLIGS SKQMRHQSES QGVGLSDKEL VSDDEERGTG
eebeebeeee eeeeeebbee eeeeeeeeee eebbeeeeeb beeeeeeeee
f                     f               f      f    f
1351       1361       1371       1381       1391
LEENNQEEQS MDSNLGEAAS GCESETSVSE DCSGLSSQSD ILTTQQRDTM
beeeeeeeee beeebeebbe eeeeeeeeee ebeebeeebe bbeeeeeeeb
                         ff        f  fff   s ffff
1401       1411       1421       1431       1441
QHNLIKLQQE MAELEAVLEQ HGSQPSNSYP SIISDSSALE DLRNPEQSTS
eeebeebeee beebeebbee eeeeeeeeee ebbeeeeeee eeeeeeeeee
f fs f fff sf  ffssf    ff
1451       1461       1471       1481       1491
EKAVLTSQKS SEYPISQNPE GLSADKFEVS ADSSTSKNKE PGVERSSPSK
eebbbeeeee eeeeeeeeee ebeeeebeee eeeeeeeeee eeeeeeeeee
     ff         f       f                         f
1501       1511       1521       1531       1541
CPSLDDRWYM HSCSGSLQNR NYPSQEELIK VVDVEEQQLE ESGPHDLTET
eebeeeebee eeeeeeeeee eeeeeeebbe ebeeeeeeee eeeeeeeeee

1551       1561       1571       1581       1591
SYLPRQDLEG TPYLESGISL FSDDPESDPS EDRAPESARV GNIPSSTSAL
eeeeeeeeee eeebeeebeb bbeeeeeeee eeeeeeeeeb bebeebebeb
        f       ff
1601       1611       1621       1631       1641
KVPQLKVAES AQSPAAAHTT DTAGYNAMEE SVSREKPELT ASTERVNKRM
ebeebebeee eeeebebebe eeeeeeeeee eeeeeeeeee eeeeebeeeb

1651       1661       1671       1681       1691
SMVVSGLTPE EFMLVYKFAR KHHITLTNLI TEETTHVVMK TDAEFVCERT
bbbbbebeee ebbbbbebbe eeebebbeeb eeebbebbbe bebebbbeeb
s sssfsf   f     fss  f          fffssfs  f sf   ssffs
1701       1711       1721       1731       1741
```

```
LKYFLGIAGG KWVVSYFWVT QSIKERKMLN EHDFEVRGDV VNGRNHQGPK
bebbbbbbbb ebbbbbbbbb ebbeeeebbe eeebebebeb bebeeeeeee
 fsssssss  f  ss   s   s ff   s  f  sfs sfs  fsfff ff 

1751       1761       1771       1781       1791
RARESQDRKI FRGLEICCYG PFTNMPTDQL EWMVQLCGAS VVKELSSFTL
ebeeeeeeeb beebebbbee eeeeeeeeeb ebbbebbebe bbeeeeebee
fsfff      s f    s f fffff ff s f    s fsf ss

1801       1811       1821       1831       1841
GTGVHPIVVV QPDAWTEDNG FHAIGQMCEA PVVTREWVLD SVALYQCQEL
eeeeebbbbb beebbeeeee beebbeebeb ebbbeebbbe bbbbbebeeb
       s    ffs            f       ssff ssf s      ff

1851       1861
DTYLIPQIPH SHY
eeebbeebee eee
  f   f
```

Legend:

The conservation scale:

1 2 3 4 5 6 7 8 9

Variable **Average** **Conserved**

e - **An exposed residue according to the neural-network algorithm.**

b - **A buried residue according to the neural-network algorithm.**

f - **A predicted functional residue (highly conserved and exposed).**

s - **A predicted structural residue (highly conserved and buried).**

X - **Insufficient data - the calculation for this site was performed on less than 10% of the sequences.**
