# Supplementary material for: Pathogenic genetic variants from highly connected cancer susceptibility genes confer the loss of structural stability
Source: Sci Rep. 2021 Sep 28;11:19264. doi: 10.1038/s41598-021-98547-y (PMC8479081; doi:10.1038/s41598-021-98547-y)
Supplement: Supplementary file 1 — Supplementary Information. [file 41598_2021_98547_MOESM1_ESM.zip › Supplementary files-5-8-21/Supplementary file 11.pdf]

ConSeq Results

```
1          11         21         31         41
MSLVLNDLLI CCRQLEHDRA TERKKEVEKF KRLIRDPETI KHLDRHSDSK
eeeebeebbb bbeeeeeeee eeeeeebeeb eebbeeeeeb eebeeeeeee
fff    s   ss  f       ff  f                sf  f

51         61         71         81         91
QGKYLNWDAV FRFLQKYIQK ETECLRIAKP NVSASTQASR QKKMQEISSL
eeeebebebb bebbeebbee eeeeeeebee ebeebeeeee eeebeebbeb
     fsf      sf    f f f          f  f   f  ff f   f

101        111        121        131        141
VKYFIKCANR RAPRLKCQEL LNYIMDTVKD SSNGAIYGAD CSNILLKDIL
bebbbebbee eeeebebeeb beebbebbee eeebbbebee bbebbbeebb
 f ss  sf  f f      s   f                    s  s f

151        161        171        181        191
SVRKYWCEIS QQQWLELFSV YFRLYLKPSQ DVHRVLVARI IHAVTKGCCS
ebeebbbebe eeebeebbeb bbebbeeeee ebbebbbbbb bbbbbebebe
fsffss       fs                     fs   s  s     s

201        211        221        231        241
QTDGLNSKFL DFFSKAIQCA RQEKSSSGLN HILAALTIFL KTLAVNFRIR
eeeeeeeebb ebbbebbeeb eeeeeeebbe bbbebbbbbb ebbbeebebe
ff           s        f              s           f f

251        261        271        281        291
VCELGDEILP TLLYIWTQHR LNDSLKEVII ELFQLQIYIH HPKGAKTQEK
bbebeeebbe bbbbbbbeee eeeebeeebb ebbebebbbe beeeeeeeee
 s  ff              f  f   f          f   f sf ff

301        311        321        331        341
GAYESTKWRS ILYNLYDLLV NEISHIGSRG KYSSGFRNIA VKENLIELMA
ebeeeeebee bbeebbebbb eebebbbbeb eeeeeeeebb beeebbebbb
fs     s               f     sfs ff    f     f       s

351        361        371        381        391
DICHQVFNED TRSLEISQSY TTTQRESSDY SVPCKRKKIE LGWEVIKDHL
ebbeebbeee eebbebeeee eeeeeeeeee eeeeeeeebe bbbebbeeeb
f sff s                              f f      s   f

401        411        421        431        441
QKSQNDFDLV PWLQIATQLI SKYPASLPNC ELSPLLMILS QLLPQQRHGE
eeeeeebebb bbbebbeebb eebeeebeee ebbebbebbe ebbeeeeeee
f    f      ssfs   s  f  f   f   f       s  f   f f  f

451        461        471        481        491
RTPYVLRCLT EVALCQDKRS NLESSQKSDL LKLWNKIWCI TFRGISSEQI
eeeebbebbe ebbebeeeee eeeeeeeeeb eebbbebbbb bbebbeeeeb
f       s    s sf                   s         f  f

501        511        521        531        541
QAENFGLLGA IIQGSLVEVD REFWKLFTGS ACRPSCPAVC CLTLALTTSI
eeebbebbbb bbeebbbebe eebbebbeee ebeeeeeebb bbbebbeebe
f     s             f               ff
```

```
551        561        571        581        591
VPGTVKMGIE QNMCEVNRSF SLKESIMKWL LFYQLEGDLE NSTEVPPILH
beeebeeeee eeeeeeeeee ebeebbbebb bbeeeeeeee eeeeeeebbb
 f
601        611        621        631        641
SNFPHLVLEK ILVSLTMKNC KAAMNFFQSV PECEHHQKDK EELSFSEVEE
eebeeebbee bbbbbbbeeb eebbebbeee eebeeeeeee eeeebeebee
            ss s  ff                                f
651        661        671        681        691
LFLQTTFDKM DFLTIVRECG IEKHQSSIGF SVHQNLKESL DRCLLGLSEQ
bbbebbbeeb eeeeeeeeee eeeeeeeeeb ebeeebeeeb eebbeebbee

701        711        721        731        741
LLNNYSSEIT NSETLVRCSR LLVGVLGCYC YMGVIAEEEA YKSELFQKAK
bbeeeeeeee eeebbbebbe bbbbbbbbbb bbbbbeeeee beeebbeebe
s                                             f     sf
751        761        771        781        791
SLMQCAGESI TLFKNKTNEE FRIGSLRNMM QLCTRCLSNC TKKSPNKIAS
ebbeebeebb eebeeeeeee eebebbeebb ebbeeebeee eeeeeeebbb

801        811        821        831        841
GFFLRLLTSK LMNDIADICK SLASFIKKPF DRGEVESMED DTNGNLMEVE
ebbbebbeee bbeebbebbe ebbeeeeeee eeeeeeeeee eeeeeeeeee

851        861        871        881        891
DQSSMNLFND YPDSSVSDAN EPGESQSTIG AINPLAEEYL SKQDLLFLDM
eeeeeebbee eeeeeeeeee eeeeeeeeee bebebbeeee eeeebebbeb
                                     s    f    f f
901        911        921        931        941
LKFLCLCVTT AQTNTVSFRA ADIRRKLLML IDSSTLEPTK SLHLHMYLML
bebbbebbee eeeeebbbee eebeeebbeb beebebebbe ebebbbbbbb
   s                        ss         f      f
951        961        971        981        991
LKELPGEEYP LPMEDVLELL KPLSNVCSLY RRDQDVCKTI LNHVLHVVKN
beebeeeeee eeeeebeebb eebeebbebe eeeeebbebb beebbebbee
                                 f ff   s    s
1001       1011       1021       1031       1041
LGQSNMDSEN TRDAQGQFLT VIGAFWHLTK ERKYIFSVRM ALVNCLKTLL
beeeeeeeee beebeeebbe bbbbbbbbee eeebebebeb bbbebbeebb
                   s      s
1051       1061       1071       1081       1091
EADPYSKWAI LNVMGKDFPV NEVFTQFLAD NHHQVRMLAA ESINRLFQDT
eeeeebebbb bebeeeeeeb eebbeebbee eeeebbbbbb ebbeebbeee
  ff                         s f   f s    s      ss
1101       1111       1121       1131       1141
KGDSSRLLKA LPLKLQQTAF ENAYLKAQEG MREMSHSAEN PETLDEIYNR
```

```
eeeeeeeeee beeeeeeebb eebbbebeee beeebeeeee eeeeeeeeee
                ff  s                           ff  ff
1151       1161       1171       1181       1191
KSVLLTLIAV VLSCSPICEK QALFALCKSV KENGLEPHLV KKVLEKVSET
ebbbbebbbb bbbbbeebee ebbbebbeeb eeeebeeebb eebbeebbee
   ss          sf sff f  sf  ff   ff         f
1201       1211       1221       1231       1241
FGYRRLEDFM ASHLDYLVLE WLNLQDTEYN LSSFPFILLN YTNIEDFYRS
bebeebeebb bebbebbbbe bbeeeeeeee beebebbbbe beebeebbee
             s   s    ss            sf            s  f
1251       1261       1271       1281       1291
CYKVLIPHLV IRSHFDEVKS IANQIQEDWK SLLTDCFPKI LVNILPYFAY
bbebbbebbb bbeebeebee bbeebeeebe ebbeebbeeb bbbbbbbbbb
      f s         s                s   sf      s s s
1301       1311       1321       1331       1341
EGTRDSGMAQ QRETATKVYD MLKSENLLGK QIDHLFISNL PEIVVELLMT
eeeeeeeebe eeeebeebbe bbeeeeeeee ebeebbbeeb eebbbebbbe
               s             fff   f     fs    ssf s f
1351       1361       1371       1381       1391
LHEPANSSAS QSTDLCDFSG DLDPAPNPPH FPSHVIKATF AYISNCHKTK
beeeeeeeee eeeebeebbe eeeeeeeeee bebbbbebbb ebbbebeeee
  f                     ff ffff  s s  s  s    s  sff
1401       1411       1421       1431       1441
LKSILEILSK SPDSYQKILL AICEQAAETN NVYKKHRILK IYHLFVSLLL
bebbbebbee eeeebbebbb bbbeebeeee ebbeebbbbb bbbbbbbbbb
       sff  f fss ss               sf  s     s  ss  ss
1451       1461       1471       1481       1491
KDIKSGLGGA WAFVLRDVIY TLIHYINQRP SCIMDVSLRS FSLCCDLLSQ
eebeeebebb bbbbbeebbb bbbbbbeeee eebeebeeeb bbbbbebbee
      sf    ssssff ss s  s  f          f fs   ss fss
1501       1511       1521       1531       1541
VCQTAVTYCK DALENHLHVI VGTLIPLVYE QVEVQKQVLD LLKYLVIDNK
bbeebbebbe ebbeebbebb bbbbbeebee eeebeeebbe bbebbbbeee
           fs   ss       s f           f    ss  s   f
1551       1561       1571       1581       1591
DNENLYITIK LLDPFPDHVV FKDLRITQQK IKYSRGPFSL LEEINHFLSV
eeeebeebbe ebeebeeeee beebeebeee beeeeeebeb eeebeebbbb
    s       sff f         f  f    ff      s   f  fsss
1601       1611       1621       1631       1641
SVYDALPLTR LEGLKDLRRQ LELHKDQMVD IMRASQDNPQ DGIMVKLVVN
bbbeebebbe beebeebeee beeeeeebee bbeebeeeee eebbbebbbe
    f    f  ff   s        f                       s
1651       1661       1671       1681       1691
LLQLSKMAIN HTGEKEVLEA VGSCLGEVGP IDFSTIAIQH SKDASYTKAL
bbebbeebee eeeeeebbee bbebbeeebe bebbbbbbee eeeebeeebb
s                  ff     sff sf    s
1701       1711       1721       1731       1741
```

```
KLFEDKELQW TFIMLTYLNN TLVEDCVKVR SAAVTCLKNI LATKTGHSFW
ebbeeeebeb bbbbbbbbee bbeeebbebe ebbbebbeeb bbeeeeeebb
                   f   s       f  s     f   s f  f
1751       1761       1771       1781       1791
EIYKMTTDPM LAYLQPFRTS RKKFLEVPRF DKENPFEGLD DINLWIPLSE
eebeeeeeeb bbbbbebeee eeebeebeee eeeebeeebe ebebbbeeee
   f   f         sf
1801       1811       1821       1831       1841
NHDIWIKTLT CAFLDSGGTK CEILQLLKPM CEVKTDFCQT VLPYLIHDIL
eeeebbeebb bbbbeeeebe eebbebbeeb bebeeebbee bbebbbbbbb
 f  s   s       f      f      f  s s     f   sf   s  s
1851       1861       1871       1881       1891
LQDTNESWRN LLSTHVQGFF TSCLRHFSQT SRSTTPANLD SESEHFFRCC
beeeeebbee bbeeebeebb ebbbeeeeee eeeeeeeeee eeeeeeeeeb
  f     f   sf      s            fff ff
1901       1911       1921       1931       1941
LDKKSQRTML AVVDYMRRQK RPSSGTIFND AFWLDLNYLE VAKVAQSCAA
eeeeeeebbb bbbebbeeee eeeeeebbee bbbbebebbe bbebbeebeb
    fff s   s f  f f  f    f   f ss s  f     s  sf  fs
1951       1961       1971       1981       1991
HFTALLYAEI YADKKSMDDQ EKRSLAFEEG SQSTTISSLS EKSKEETGIS
bbbbbbbbeb bbeeeeeeee eeeebebeee eeebebbebe eeeeeeeebb
ssss ss fs   f                   ff       f   f
2001       2011       2021       2031       2041
LQDLLLEIYR SIGEPDSLYG CGGGKMLQPI TRLRTYEHEA MWGKALVTYD
beebbbebbe bbeeeeebeb beeeeeeeeb bebeebeeeb ebeebbbbbe
 f  s   s  ssfffffsfs  f f        f ffsfff     fss ssf
2051       2061       2071       2081       2091
LETAIPSSTR QAGIIQALQN LGLCHILSVY LKGLDYENKD WCPELEELHY
beeebeebbe ebbbbebbee bebbbbbbbb beebeeeeee bbeebeebbb
         f   s     ff  fs   s  s   fs          f  f
2101       2111       2121       2131       2141
QAAWRNMQWD HCTSVSKEVE GTSYHESLYN ALQSLRDREF STFYESLKYA
ebbbeebebe eeeeeeeeee eeeeeebbbe bbeebbeeeb eebeebbeeb
f s f                   f f         fs f f
2151       2161       2171       2181       2191
RVKEVEEMCK RSLESVYSLY PTLSRLQAIG ELESIGELFS RSVTHRQLSE
eeeebeebbe ebbeebbebb ebbeebeebe ebeebeebbe eebeeeebee
f     fs     sff  f s f s  sf    f
2201       2211       2221       2231       2241
VYIKWQKHSQ LLKDSDFSFQ EPIMALRTVI LEILMEKEMD NSQRECIKDI
bbeebeeebe bbeebeeebe bbbbbbbbbb bebbbeeeee eeeeebbeeb
                ff    ss    s
2251       2261       2271       2281       2291
LTKHLVELSI LARTFKNTQL PERAIFQIKQ YNSVSCGVSE WQLEEAQVFW
beeebeebbe bbeeeeeeeb beebbbebee eeeeeeeebe bebeebebbb
   fs       sf f f f  sf s    f               sffsf  s
```

```
2301       2311       2321       2331       2341
AKKEQSLALS ILKQMIKKLD ASCAANNPSL KLTYTECLRV CGNWLAETCL
eeeeeebbbe bbeebbeebe eebeeeeeeb eebbbebbeb beebbbebbe
 f f   s           s                   sf   f  s f
2351       2361       2371       2381       2391
ENPAVIMQTY LEKAVEVAGN YDGESSDELR NGKMKAFLSL ARFSDTQYQR
eeeebbbeeb beebbebbee eeeeeeeeee eeebebbbbb bebeeeebee
fff  s      f                     f   s sss sfsff fsf
2401       2411       2421       2431       2441
IENYMKSSEF ENKQALLKRA KEEVGLLREH KIQTNRYTVK VQRELELDEL
beebbeeeeb eeeeebbeeb eeebeebeee eeeeeebbbe beeeeeeeee
s f s fff  fff          f  f          fs    s  f   f
2451       2461       2471       2481       2491
ALRALKEDRK RFLCKAVENY INCLLSGEEH DMWVFRLCSL WLENSGVSEV
bbeebeeeee ebbbebbbeb bebbeeeeee ebbbbbbbbb bbeeeebeeb
s      f    ss fs  fs   ss  f  f f   ss  ss ss f
2501       2511       2521       2531       2541
NGMMKRDGMK IPTYKFLPLM YQLAARMGTK MMGGLGFHEV LNNLISRISM
eebbeeebee beebebbebb bebbeebbee eeeebbbeeb beebbbebbe
f          s f fssfs  sfssffs ff       s    s  s
2551       2561       2571       2581       2591
DHPHTLFFII LALANANRDE FLTKPEVARR SRITKNVPKQ SSQLDEDRTE
ebeebbbbbb bbbbeeeeee eeeeeeeeee eebeeeeeee eeeeeeeebe
 sffs s ss  ss fffff                         f  f  f
2601       2611       2621       2631       2641
AANRIICTIR SRRPQMVRSV EALCDAYIIL ANLDATQWKT QRKGINIPAD
bbeebbebbe eeeeebbeeb eebbebbbbb bbbeeeeeee eeeebebeee
 s                    f s  sss   s  ff       f    sf
2651       2661       2671       2681       2691
QPITKLKNLE DVVVPTMEIK VDHTGEYGNL VTIQSFKAEF RLAGGVNLPK
eebeebeebe ebbbebbebe beeeeeeeeb bbbebbeeeb ebeebbebee
ff          s  fs     sf ff f f       s      s fs f ff
2701       2711       2721       2731       2741
IIDCVGSDGK ERRQLVKGRD DLRQDAVMQQ VFQMCNTLLQ RNTETRKRKL
bbebebeeee eeeeebeeee ebeeebbbee bbebbbebbe eeeeeeeeeb
ssfs sfff   ffffsff f fsfffsssff ss sss ss   f fff ffs
2751       2761       2771       2781       2791
TICTYKVVPL SQRSGVLEWC TGTVPIGEFL VNNEDGAHKR YRPNDFSAFQ
ebeebebbeb eeeeebbebb eeebebbebb beeeeeeeee beeeebeebe
 sffsfs f  fffff sfss  ff f s  s      fff f  ff f
2801       2811       2821       2831       2841
CQKKMMEVQK KSFEEKYEVF MDVCQNFQPV FRYFCMEKFL DPAIWFEKRL
beeebeebee eebeeebeeb eebbeebeeb bbbbbbeebe eebbbbeebb
sf  s           f           s fs s  s  ffs  ffs s f  s
2851       2861       2871       2881       2891
AYTRSVATSS IVGYILGLGD RHVQNILINE QSAELVHIDL GVAFEQGKIL
```

```
ebbebbbbbb bbbbbbbbbb ebbbbbbbee ebeebbbbeb bbbbeebeee
fssfssssss sssss ssss fs ssss f    ffs ssfs ssssffsfff
2901       2911       2921       2931       2941
PTPETVPFRL TRDIVDGMGI TGVEGVFRRC CEKTMEVMRN SQETLLTIVE
eeeeebebeb beebbbbbbb bbbeebbeeb beebbebbee beebbbebbe
fffffsfsfs  ff sssss   ssffssffs sffss ssf  sff   fssf
2951       2961       2971       2981       2991
VLLYDPLFDW TMNPLKALYL QQRPEDETEL HPTLNADDQE CKRNLSDIDQ
bbbbbbbbeb beeebebbeb eeeeeeeeeb ebebeeeeee eeeeeeeeee
sss sss fs sfff fs  s f    f       f                 f
3001       3011       3021       3031       3041
SFNKVAERVL MRLQEKLKGV EEGTVLSVGG QVNLLIQQAI DPKNLSRLFP
ebeebbeebb eebeeebeee eeeebbebeb ebbbbbeebe eeeebeebbe
f ffssffss  fsfffsfff f f ssfs s fss ssffs  ff fsf s
3051
GWKAWV
ebeeee
fsf  f
```

Legend:

The conservation scale:

1 2 3 4 5 6 7 8 9

Variable **Average** **Conserved**

e - **An exposed residue according to the neural-network algorithm.**
b - **A buried residue according to the neural-network algorithm.**
f - **A predicted functional residue (highly conserved and exposed).**
s - **A predicted structural residue (highly conserved and buried).**
X - **Insufficient data - the calculation for this site was performed on less than 10% of the sequences.**
