# Supplementary material for: Pathogenic genetic variants from highly connected cancer susceptibility genes confer the loss of structural stability
Source: Sci Rep. 2021 Sep 28;11:19264. doi: 10.1038/s41598-021-98547-y (PMC8479081; doi:10.1038/s41598-021-98547-y)
Supplement: Supplementary file 1 — Supplementary Information. [file 41598_2021_98547_MOESM1_ESM.zip › Supplementary files-5-8-21/Supplementary file 12.pdf]

ConSeq Results

```
1          11         21         31         41
MEEPQSDPSV EPPLSQETFS DLWKLLPENN VLSPLPSQAM DDLMLSPDDI
eeeeeeeeee eeeeeeeebe ebbeebeeee ebeeebeeeb eebbebeeeb
f            f

51         61         71         81         91
EQWFTEDPGP DEAPRMPEAA PRVAPAPAAP TPAAPAPAPS WPLSSSVPSQ
eeebeeeeee eebeeeeeee eeeeeeeeee eeeeeeeeee eeeeeebeee
                                                   f

101        111        121        131        141
KTYQGSYGFR LGFLHSGTAK SVTCTYSPAL NKMFCQLAKT CPVQLWVDST
eeeeeeeebe bebeeeeeee eeebeebeeb eebbbebbee bebebebeee
 f  f        s  f fff fff f s  s ffs sf  ff  f

151        161        171        181        191
PPPGTRVRAM AIYKQSQHMT EVVRRCPHHE RCSDSDGLAP PQHLIRVEGN
eeeebbbbbb bbbeeeeebe ebbeebeeee eeeeeeeeee eebbbebeee
ff f   ss  s  ff ff f  ss fsffff          f   sssf ff

201        211        221        231        241
LRVEYLDDRN TFRHSVVVPY EPPEVGSDCT TIHYNYMCNS SCMGGMNRRP
eebeeeeeee eeeebbbbee eeeeeeeebb bbbbbbbbee ebeebeeeeb
    f  f   f f ss  ff f ff f     s s   ssff fsffsffff

251        261        271        281        291
ILTIITLEDS SGNLLGRNSF EVHVCACPGR DRRTEEENLR KKGEPHHELP
bbbbbbbeee eeebbeeebb ebebbbbeee eeeeeeeeee eeeeeeeeee
s  s ssf    f  sfff s fsf sssfff fff ff

301        311        321        331        341
PGSTKRALSN NTSSSPQPKK KPLDGEYFTL QIRGRERFEM FRELNEALEL
eeeeeeeeee eeeeeeeeee eeeeeebbbb ebeeeeebeb beebeebbeb
    ff             f                ff         f   f

351        361        371        381        391
KDAQAGKEPG GSRAHSSHLK SKKGQSTSRH KKLMFKTEGP DSD
eeeeeeeeee eeeeeeeeee eeeeeeeeee eebebeeeee eee
                                 f    f f   fff
```

Legend:

The conservation scale:

1 2 3 4 5 6 7 8 9

Variable **Average** **Conserved**

e - An exposed residue according to the neural-network algorithm.

b - A buried residue according to the neural-network algorithm.

f - A predicted functional residue (highly conserved and exposed).

s - A predicted structural residue (highly conserved and buried).

X - Insufficient data - the calculation for this site was performed on less than 10% of the sequences.
