# Supplementary material for: Pathogenic genetic variants from highly connected cancer susceptibility genes confer the loss of structural stability
Source: Sci Rep. 2021 Sep 28;11:19264. doi: 10.1038/s41598-021-98547-y (PMC8479081; doi:10.1038/s41598-021-98547-y)
Supplement: Supplementary file 1 — Supplementary Information. [file 41598_2021_98547_MOESM1_ESM.zip › Supplementary files-5-8-21/Supplementary file 16.docx]

| **miR ID** | **dbSNP ID** | **miR Seed** | **miRSite** | **Conservation** | **Context+ score change** |
| --- | --- | --- | --- | --- | --- |
| hsa-miR-4311 | rs142080198 | AA[AG/-]GAGA | UCUCUUU | 2 | -0.111 |
| hsa-miR-3910 | rs149611497 | AAGGCA[GGCAT/-] | AUGCCUU | 2 | -0.038 |
| hsa-miR-3161 | rs113098367 | UG[-/A]UAAG | UUAUCAA | 2 | 0.055 |
| hsa-miR-3161 | rs11382316 | UGAU[-/A]AG | UUAUCAA | 2 | 0.055 |
| hsa-miR-4525 | rs34711227 | GGGGG[-/G]U | UCCCCCA | 2 | -0.07 |
| hsa-miR-1276 | rs34381260 | AAAG[-/A]GC | CUCUUUA | 2 | -0.017 |
| hsa-miR-4274 | rs202195689 | AGCAG[-/CCC]C | GACUGCU | 2 | -0.219 |
| hsa-miR-4301 | rs184176277 | [C/A]CCACUA | UAGUGGG | 3 | -0.032 |
| hsa-miR-645 | rs201355281 | [C/G]UAGGCU | AGCCUAG | 2 | -0.179 |
| hsa-miR-6509-3p | rs145322812 | UC[C/T]ACUG | AGUGGAA | 3 | 0.008 |
| hsa-miR-6796-3p | rs3745198 | AAGCU[C/G]U | AGAGCUU | 5 | -0.025 |
| hsa-miR-3655 | rs146400503 | CUUGU[C/T]G | GACAAGA | 2 | -0.054 |
| hsa-miR-642b-3p | rs111664333 | GA[C/T]ACAU | AUGUGUC | 3 | -0.057 |
| hsa-miR-5007-5p | rs191336981 | [A/G]GAGUCU | AGACUCU | 2 | -0.06 |
| hsa-miR-3675-5p | rs202014433 | AUGG[G/A]GC | CCCCAUA | 2 | -0.037 |
| hsa-miR-3622b-5p | rs13276615 | [G/T]GCAUGG | CAUGCCA | 2 | -0.101 |
| hsa-miR-548ap-5p | rs186097449 | AAA[G/C]UAA | UUACUUU | 3 | -0.116 |
| hsa-miR-548ao-3p | rs79091838 | AAGAC[C/T]G | GGUCUUA | 2 | -0.189 |
| hsa-miR-5197-3p | rs77549240 | AGAAGA[G/T] | CUCUUCU | 2 | 0.019 |
| hsa-miR-4781-3p | rs74085143 | AU[G/A]UUGG | CCAACAUA | 2 | -0.297 |
| hsa-miR-936 | rs79924817 | C[A/G]GUAGA | CUACUGA | 3 | 0.026 |
| hsa-miR-8073 | rs200984263 | CCU[G/T]GCA | GCCAGGA | 2 | -0.026 |
| hsa-miR-221-5p | rs113054794 | CCU[G/T]GCA | GCCAGGA | 2 | -0.026 |
| hsa-miR-5007-3p | rs184597603 | UCAU[A/G]UG | AUAUGAA | 4 | No Change |
| hsa-miR-5007-3p | rs12865044 | UCAUAU[G/C] | AUAUGAA | 4 | No Change |
| hsa-miR-6810-5p | rs62182086 | UGGGG[A/G]C | UCCCCAA | 2 | -0.151 |
| hsa-miR-6810-5p | rs74718922 | UGGGGA[C/T] | UCCCCAA | 2 | -0.151 |
| hsa-miR-450a-1-3p | rs185256782 | UUG[G/C]GAA | UCCCCAAA | 2 | -0.19 |
| hsa-miR-450a-2-3p | rs185256782 | UUG[G/C]GGA | UCCCCAAA | 2 | -0.19 |
| hsa-miR-6874-3p | rs187466447 | [A/G]GUUCUG | CAGAACU | 4 | 0.01 |

**Supplementary Table S1:** Target sites created by SNPs and INDELs of ATM in miRNA seeds

| **miR ID** | **dbSNP ID** | **miR Seed** | **miRSite** | **Conservation** | **Context+**  **score change** |
| --- | --- | --- | --- | --- | --- |
| hsa-miR-6777-3p | rs191900683 | CCACU[C/T]U | AAGUGGA | 3 | 0.033 |
| hsa-miR-6814-5p | rs185472832 | CCCAA[G/A]G | CUUUGGGA | 2 | -0.188 |
| hsa-miR-6767-5p | rs113675906 | [C/T]GCAGAC | UCUGCAA | 2 | -0.05 |
| hsa-miR-3126-5p | rs182148652 | [G/A]AGGGAC | GUCCCAC | 2 | -0.02 |
| hsa-miR-3126-5p | rs187256070 | G[A/T]GGGAC | GUCCCAC | 2 | -0.02 |
| hsa-miR-4743-5p | rs141766192 | GGC[C/T]GGA | UCCAGCC | 2 | -0.179 |
| hsa-miR-8065 | rs190290362 | GU[A/G]GGAA | UUCCCAC | 2 | -0.181 |
| hsa-miR-4788 | rs187884409 | UAC[G/A]GAC | GUCUGUA | 2 | -0.152 |
| hsa-miR-4540 | rs143699849 | UAGUC[C/T]U | AAGACUA | 2 | -0.222 |
| hsa-miR-6788-3p | rs115219828 | U[C/T]GCCAC | UGGCAAA | 2 | 0.028 |
| hsa-miR-488-5p | rs199722070 | CCA[G/A]AUA | UAUUUGGA | 2 | 0.093 |
| hsa-miR-488-5p | rs186200318 | CC[A/G]GAUA | UAUUUGGA | 2 | 0.093 |
| hsa-miR-6769b-3p | rs201952639 | CCUCU[C/T]U | AAAGAGGA | 4 | -0.288 |
| hsa-miR-4315 | rs143523766 | C[G/A]CUUUC | AAAGCAA | 3 | -0.241 |
| hsa-miR-4315 | rs147167801 | [C/T]GCUUUC | AAAGCAA | 3 | -0.241 |
| hsa-miR-5090 | rs3823658 | C[G/A]GGGCA | GCCCUGA | 2 | -0.108 |
| hsa-miR-8088 | rs144259950 | CUC[G/A]GUA | ACUGAGA | 2 | -0.03 |
| hsa-miR-8088 | rs181345830 | CU[C/T]GGUA | ACUGAGA | 2 | -0.03 |
| hsa-miR-15b-3p | rs192595529 | [G/A]AAUCAU | UGAUUUA | 4 | -0.026 |
| hsa-miR-6781-5p | rs113788577 | [G/A]GGCCGG | CCGGCCU | 2 | -0.044 |
| hsa-miR-505-3p | rs143213653 | [G/A]UCAACA | UGUUGAU | 3 | -0.023 |
| hsa-miR-188-3p | rs191840972 | UCCCA[C/T]A | UAUGGGAA | 2 | -0.091 |
| hsa-miR-3156-3p | rs112428304 | U[C/T]CCACU | AGUGGAA | 3 | No Change |
| hsa-miR-6727-5p | rs202175375 | U[C/T]GGGGC | CCCCAAA | 4 | -0.069 |
| hsa-miR-525-3p | rs190453265 | AAGG[C/T]GC | GCACCUUA | 7 | -0.438 |
| hsa-miR-575 | rs149186367 | AG[C/T]CAGU | ACUGACU | 4 | 0.011 |
| hsa-miR-3689d | rs143142996 | GGAGGU[G/A] | UACCUCC | 2 | -0.19 |
| hsa-miR-4759 | rs140724460 | A[G/A]GACUA | UAGUCUU | 6 | -0.203 |
| hsa-miR-1248 | rs202149708 | CCUU[C/T]UU | AAAAAGG | 2 | -0.145 |
| hsa-miR-3117-3p | rs12402181 | UA[G/A]GACU | GUCUUAA | 2 | -0.086 |
| hsa-miR-4731-3p | rs66507245 | ACACA[A/T]G | CAUGUGU | 3 | 0.066 |

**Supplementary Table S2:** Target sites created by SNPs and INDELs of ATM in miRNA seeds

| **miR ID** | **dbSNP ID** | **miR Seed** | **miRSite** | **Conservation** | **Context+**  **score change** |
| --- | --- | --- | --- | --- | --- |
| hsa-miR-518e-5p | rs74177813 | U[C/-]UAGAG | UCUAGAA | 2 | No Change |
| hsa-miR-518e-5p | rs34416818 | UCU[-/A]GAG | UCUAGAA | 2 | No Change |
| hsa-miR-518e-5p | rs74177814 | UCUA[-/G]AG | UCUAGAA | 2 | No Change |
| hsa-miR-1233-5p | rs71309450 | GU[-/G]GGAG | UCCCACA | 5 | -0.192 |
| hsa-miR-125a-5p | rs12975333 | CCCUGA[G/T] | CUCAGGG | 5 | -0.22 |
| hsa-miR-3126-5p | rs182148652 | [G/A]AGGGAC | GUCCCUCA | 2 | -0.369 |
| hsa-miR-3126-5p | rs187256070 | G[A/T]GGGAC | GUCCCUCA | 2 | -0.369 |
| hsa-miR-1324 | rs79101433 | [C/T]AGACAG | UGUCUGA | 4 | -0.207 |
| hsa-miR-1324 | rs111606583 | [C/T]AGACAG | UGUCUGA | 4 | -0.207 |
| hsa-miR-1324 | rs199975935 | [C/T]AGACAG | UGUCUGA | 4 | -0.207 |
| hsa-miR-1324 | rs113859132 | CAGA[C/T]AG | UGUCUGA | 4 | -0.207 |
| hsa-miR-6754-5p | rs113246861 | CAG[G/A]GAG | CUCCCUG | 2 | -0.121 |
| hsa-miR-488-5p | rs199722070 | CCA[G/A]AUA | AUCUGGA | 2 | -0.098 |
| hsa-miR-488-5p | rs186200318 | CC[A/G]GAUA | AUCUGGA | 2 | -0.098 |
| hsa-miR-4305 | rs138201016 | CUAGA[C/A]A | GUCUAGA | 2 | -0.086 |
| hsa-miR-4305 | rs149597993 | CUAG[A/G]CA | GUCUAGA | 2 | -0.086 |
| hsa-miR-4305 | rs4636784 | CUA[G/C]ACA | GUCUAGA | 2 | -0.086 |
| hsa-miR-4271 | rs189148716 | G[G/C]GGAAG | CUUCCCC | 2 | -0.331 |
| hsa-miR-3622b-5p | rs13276615 | [G/T]GCAUGG | CAUGCCA | 2 | -0.092 |
| hsa-miR-4482-5p | rs45596840 | A[C/T]CCAGU | ACUGGGU | 2 | -0.161 |
| hsa-miR-940 | rs149527765 | A[G/A]GCAGG | CCUGCCU | 2 | -0.125 |
| hsa-miR-6742-3p | rs138408187 | CCUGG[G/A]U | ACCCAGGA | 5 | -0.332 |
| hsa-miR-6777-5p | rs56155608 | [C/T]GGGGAG | CUCCCCG | 2 | -0.186 |
| hsa-miR-6808-5p | rs200363718 | [A/G]GGCAGG | CCUGCCU | 2 | -0.115 |

**Supplementary Table S3:** Target sites disrupted by SNPs and INDELs in miRNA seeds

| **miR ID** | **dbSNP ID** | **miR Seed** | **miRSite** | **Conservation** | **Context+**  **score change** |
| --- | --- | --- | --- | --- | --- |
| hsa-miR-6814-5p | rs185472832 | CCCAA[G/A]G | UUUGGGA | 5 | -0.065 |
| hsa-miR-6767-5p | rs113675906 | [C/T]GCAGAC | UCUGCAA | 5 | -0.091 |
| hsa-miR-4738-3p | rs144216044 | GAAA[C/T]UG | CAAUUUC | 5 | -0.209 |
| hsa-miR-6859-3p | rs71260065 | GACCC[C/T]C | AGGGUCA | 4 | -0.189 |
| hsa-miR-6859-3p | rs80356110 | GACCC[C/T]C | AGGGUCA | 4 | -0.189 |
| hsa-miR-6859-3p | rs201535981 | GACCC[C/T]C | AGGGUCA | 4 | -0.189 |
| hsa-miR-938 | rs12416605 | [G/A]CCCUUA | UAAGGGU | 3 | -0.248 |
| hsa-miR-6791-3p | rs200895660 | [G/T]CCUCCU | AGGAGGA | 2 | -0.054 |
| hsa-miR-4743-5p | rs141766192 | GGC[C/T]GGA | CCAGCCA | 2 | -0.115 |
| hsa-miR-6729-5p | rs75036690 | GGGC[G/A]AG | UUGCCCA | 2 | -0.216 |
| hsa-miR-8065 | rs190290362 | GU[A/G]GGAA | UCCCACA | 5 | -0.086 |
| hsa-miR-8088 | rs144259950 | CUC[G/A]GUA | ACCAAGA | 4 | -0.067 |
| hsa-miR-8088 | rs181345830 | CU[C/T]GGUA | ACCAAGA | 4 | -0.067 |
| hsa-miR-146a-3p | rs2910164 | CU[C/G]UGAA | UCACAGA | 3 | -0.061 |
| hsa-miR-6763-3p | rs3751304 | UCCC[C/T]GG | CCAGGGAA | 7 | -0.238 |
| hsa-miR-6796-3p | rs3745198 | AAGCU[C/G]U | CAGCUUA | 4 | -0.125 |
| hsa-miR-8078 | rs77583254 | GUCU[A/T]GG | CCAAGAC | 4 | -0.16 |
| hsa-miR-6892-5p | rs111347704 | U[A/G]AGGGA | UCCCUCA | 2 | -0.139 |
| hsa-miR-3125 | rs200914693 | AG[A/G]GGAA | UUCCCCU | 2 | -0.158 |
| hsa-miR-548t-3p | rs73872515 | AAA[A/C]CCA | UGGGUUU | 2 | -0.15 |
| hsa-miR-548i | rs201549145 | AAA[G/A]UAA | UUAUUUUA | 11 | 0.014 |
| hsa-miR-1248 | rs202149708 | CCUU[C/T]UU | AAAAGGA | 3 | -0.055 |
| hsa-miR-604 | rs186844507 | GGCUGC[G/A] | UGCAGCC | 2 | -0.164 |
| hsa-miR-3199 | rs200986762 | [G/A]GGACUG | CAGUCCU | 2 | -0.053 |
| hsa-miR-4296 | rs182347826 | UGUGG[G/T]C | GACCACA | 2 | -0.064 |

**Supplementary Table S4:** Target sites created by SNPs and INDELs in miRNA seeds

| **Protein** | **Residue** | **Structure** | **Phi angle** | **Psi angle** | **Solvent accessible area** |
| --- | --- | --- | --- | --- | --- |
| WT BRCT | 1687 (VAL) | Strand | 122.59 | 117.10 | 0.4 |
|  | 1736 (VAL) | Coil | -74.95 | 151.84 | 1.0 |
| BRCT Mutants | 1687 (GLY) | Strand | 120.52 | 115.57 | 10.9 |
|  | 1736 | Coil | -73.45 | 151.29 | 14.5 |
| WT Catalytic domain | 2865 (ILE) | Alpha Helix | -64.42 | -45.15 | 26.0 |
|  | 2906 | Coil | 155.79 | 159.83 | 60.5 |
| Catalytic domain mutants | 2865 | Alpha Helix | -62.43 | -45.24 | 25.7 |
|  | 2906 | Coil | 155.20 | 158.24 | 42.3 |
| WT DBD | 216 | Strand | 124.68 | 132.40 | 0.7 |
|  | 194 | Coil | -71.98 | -47.01 | 5.0 |
| DBD mutants | 216 | Strand | 122.51 | 130.93 | 3.1 |
|  | 194 | Coil | -72.61 | -46.17 | 4.8 |

**Supplementary Table S5**: Changes in secondary structure elements analyzed from Stride server.

| **Protein** | **Molecular weight** | **Theoretical pI** | **Ext. coefficient (in**  **M−1 cm−1)** | **Est. half-life (in**  **mammalian cell)** | **Instability index** | **Aliphatic index** |
| --- | --- | --- | --- | --- | --- | --- |
| WT-BRCT | 24160.82 | 5.81 | 36815 | 1 hour | 25.33 | 83.93 |
| V1687G | 24118.74 | 5.81 | 36815 | 1 hour | 24.84 | 82.56 |
| V1736G | 24118.74 | 5.81 | 36815 | 1 hour | 25.33 | 82.56 |
| WT-Catalytic domain | 32308.41 | 7.65 | 27430 | 20 hours | 42.93 | 84.82 |
| I2865T | 32296.36 | 7.65 | 27430 | 20 hours | 41.94 | 83.43 |
| V2906A | 32280.36 | 7.65 | 27430 | 20 hours | 42.93 | 84.14 |
| WT-DBD | 23211.26 | 8.70 | 18045 | 3.5 hours | 69.67 | 62.68 |
| V216G | 23169.18 | 8.70 | 18045 | 3.5 hours | 69.67 | 61.27 |
| L194H | 23235.24 | 8.70 | 18045 | 3.5 hours | 71.82 | 60.78 |

**Supplementary Table S6**: Physicochemical properties of WT and mutant proteins analyzed from ProtParam server.

| **WT and Mutant proteins** | **Common residues** |
| --- | --- |
| WT-V1687 and mutant-V1687G | Gly1687 (WT), Val1687 (mutant), Lys1702 and Val1713 |
| WT-V1736 and mutant-V1736G | Met1689, Cys1697, Ile1707, Val1736 (WT), Gly1736 (mutant), Pro1749 |
| WT-I2865 and mutant-I2865T | Ile183 (WT), Thr183 (mutant), Phe212, Met253, Gln260 and Leu263 |
| WT-V2906 and mutant-V2906 | Val224 (WT) and Ala224 (mutant) |
| WT-V216 and mutant-V216G | Ala159, Ile195, Tyr205, His214, Val216 (WT) and Gly216 (mutant) |
| WT-L194 and mutant-L194H | Ala161, Val173, Arg174, Leu194 (WT) and His194 (mutant) His214 and Val216 |

**Supplementary Table S7**: Common residues of interactions in WT and mutants with PK083.

| **System** | **Number of solvent molecules** | **Number of Na ions** | **Number of Cl ions** |
| --- | --- | --- | --- |
| WT-BRCT | 8929 | 17 | 22 |
| V1687G | 8936 | 17 | 22 |
| V1736G | 8925 | 17 | 22 |
| WT-Catalytic domain | 11389 | 23 | 24 |
| I2865T | 11346 | 23 | 24 |
| V2906A | 11347 | 23 | 24 |
| WT-DBD | 9025 | 17 | 22 |
| V216G | 9051 | 17 | 22 |
| L194H | 9047 | 17 | 22 |
| V1687G-PK083 | 8936 | 17 | 22 |
| V1736G-PK083 | 8925 | 17 | 22 |
| I2865T-PK083 | 11346 | 23 | 24 |
| V2906A-PK083 | 11347 | 23 | 24 |
| V216G-PK083 | 9051 | 17 | 22 |
| L194H-PK083 | 9047 | 17 | 22 |

**Supplementary table S8**: The number of solvent molecules, Na, and Cl ions added to each system during MD simulation.


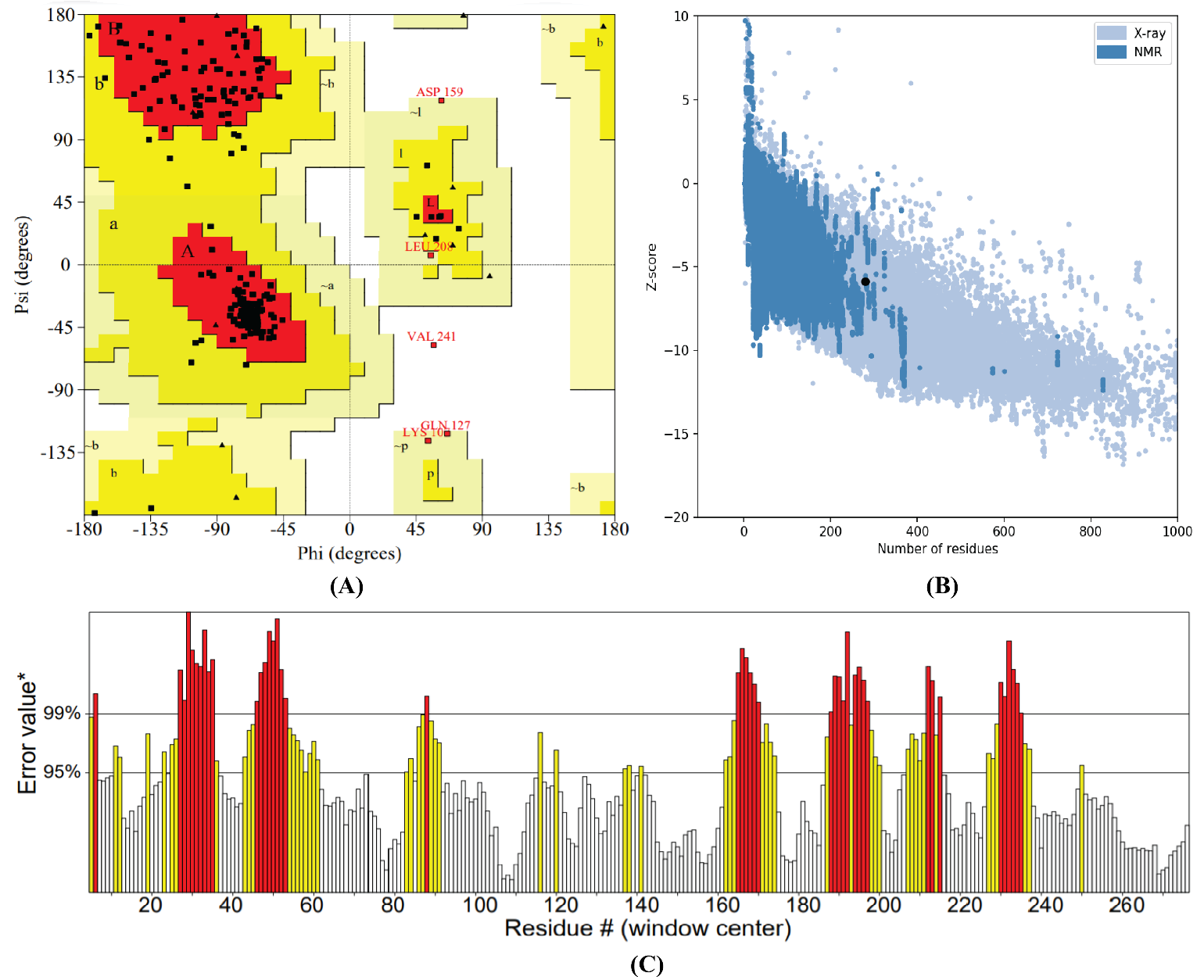


**Supplementary Figure S1**: 3D structure validation results of catalytic domain of ATM. (A) Ramachandran plot of modelled structure validated by PROCHECK program, here 225 (90.0%) residues occur in most favoured regions. (B) Validation by ProSA-web server, here, the calculated Z-score of the protein is -5.87. (C) Validation by ERRAT server, here the overall quality factor of the protein is 63.971.


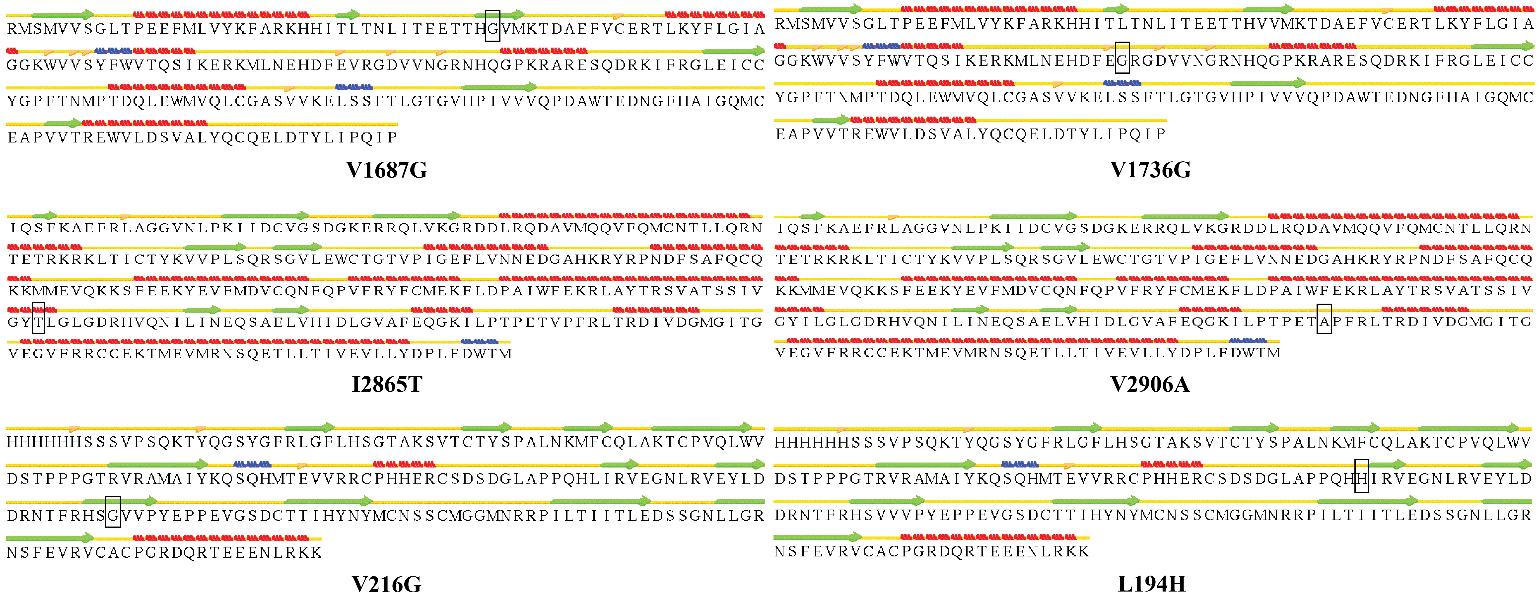


**Supplementary Figure S2:** Graphical representation of mutation location in the secondary structure elements analyzed from Stride server.


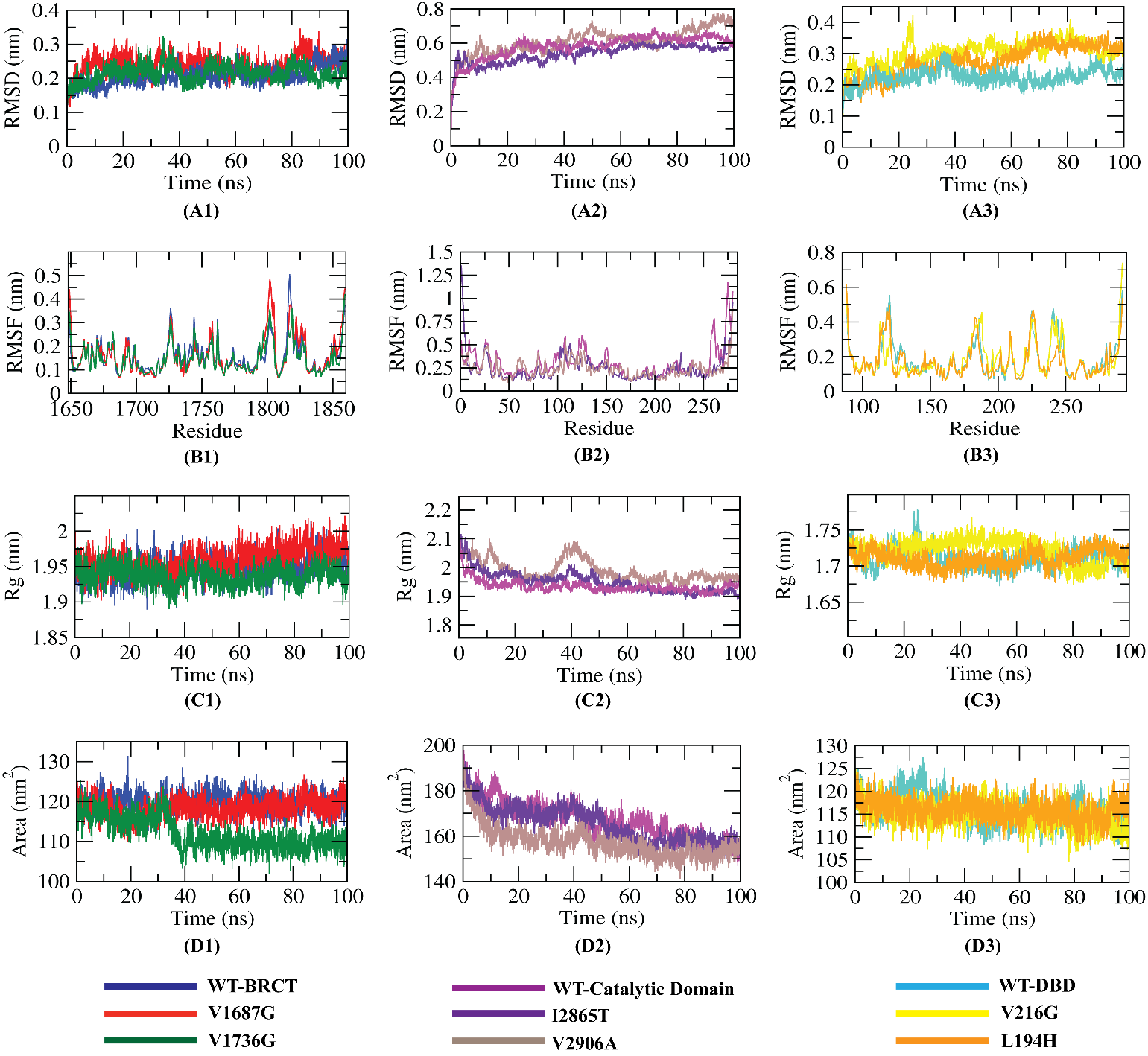


**Supplementary Figure S3:** MD simulation results (2^nd^ run) of WT and mutant variants. (A1-A3) RMSD analysis, (B1-B3) RMSF analysis, (C1-C3) radius of gyration (Rg) analysis, and (D1-D3) SASA analysis.


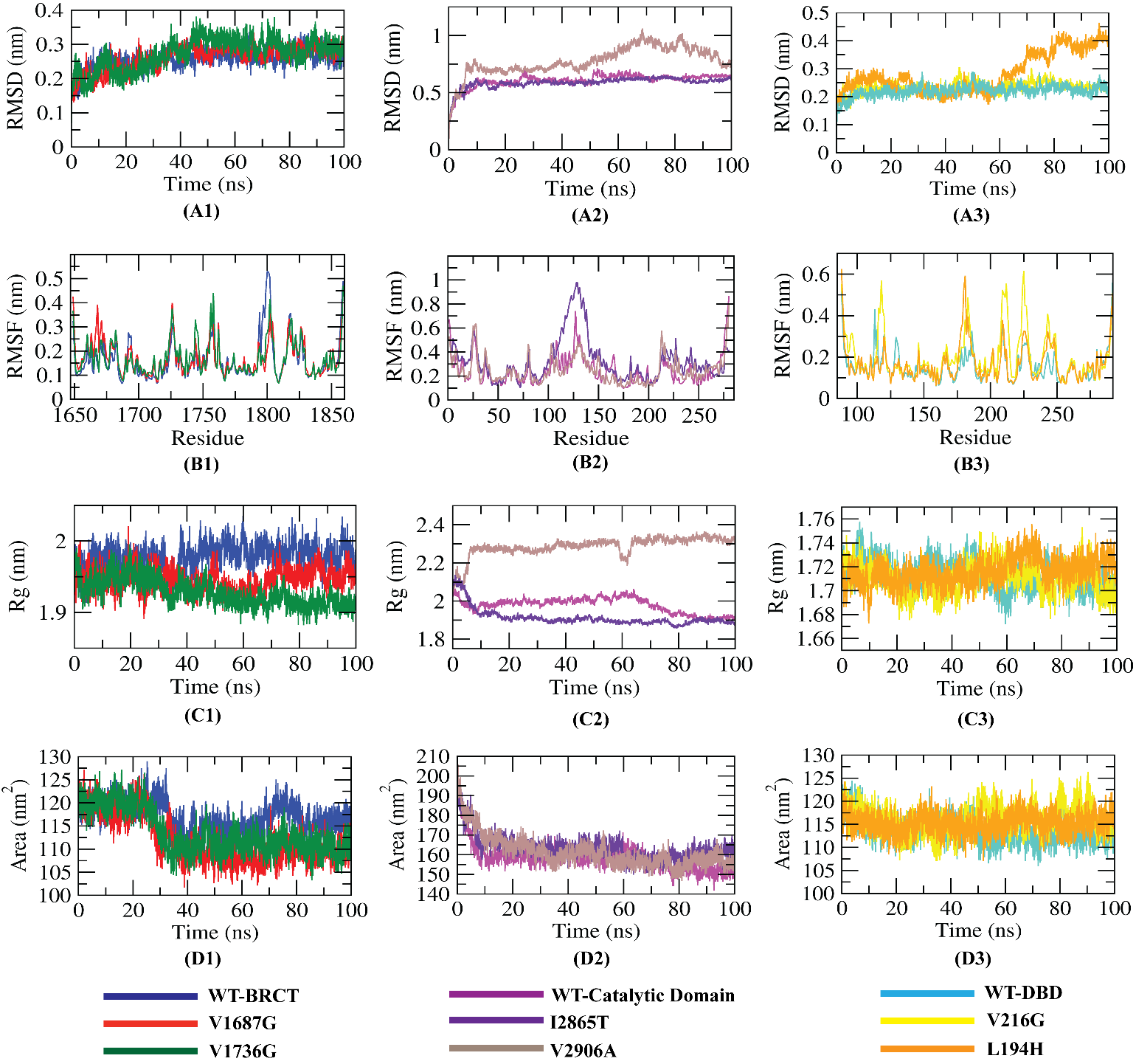


**Supplementary Figure S4:** MD simulation results (3^rd^ run) of WT and mutant variants. (A1-A3) RMSD analysis, (B1-B3) RMSF analysis, (C1-C3) radius of gyration (Rg) analysis, and (D1-D3) SASA analysis.


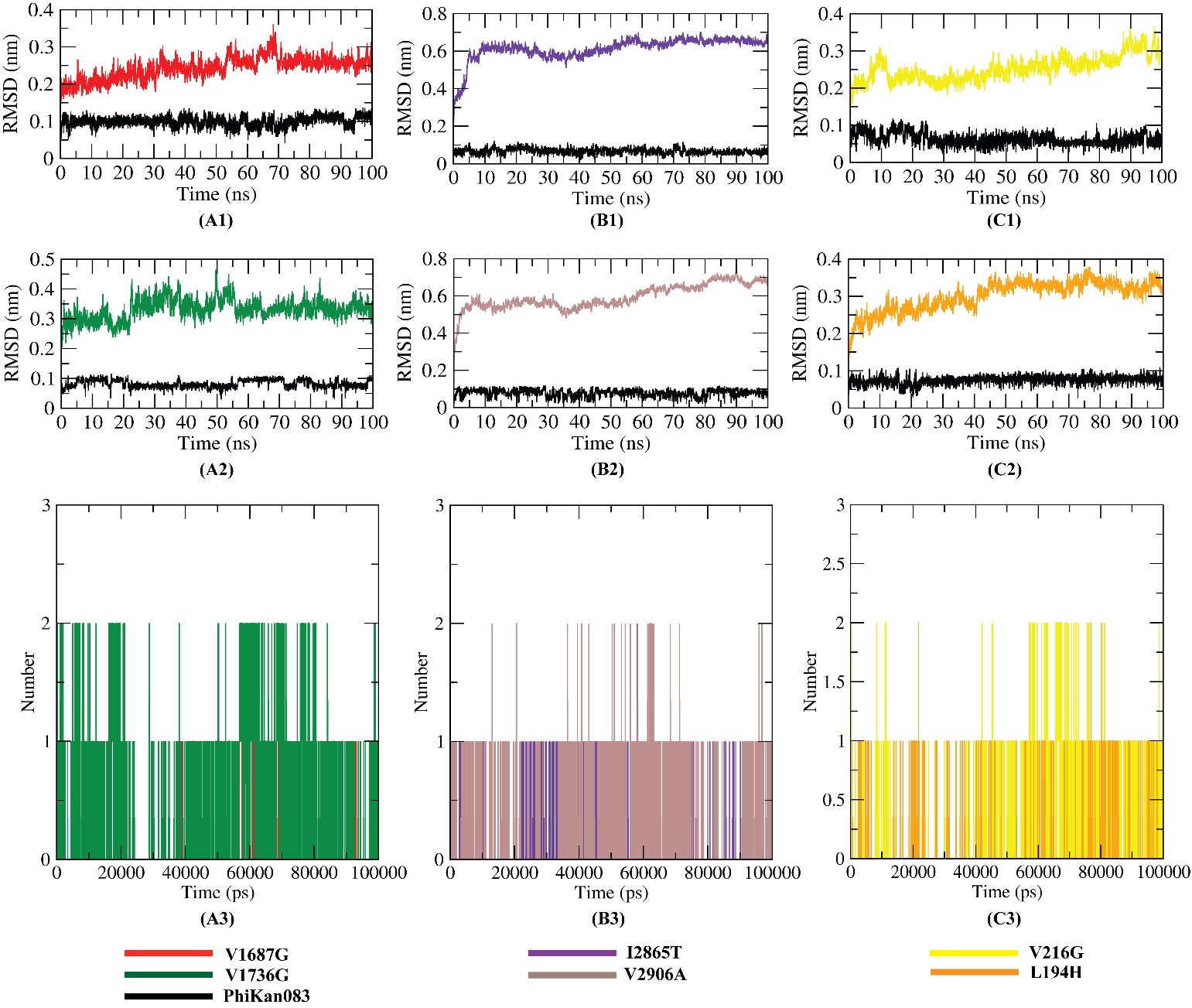


**Supplementary Figure S5:** MD simulation results (2^nd^ run) of mutant-PK083 complexes. (A1-C2) RMSD of PK083 and protein backbone obtained from 100 ns MD simulation. (A3-C3) Hydrogen bond analysis between PK083 and the mutants.


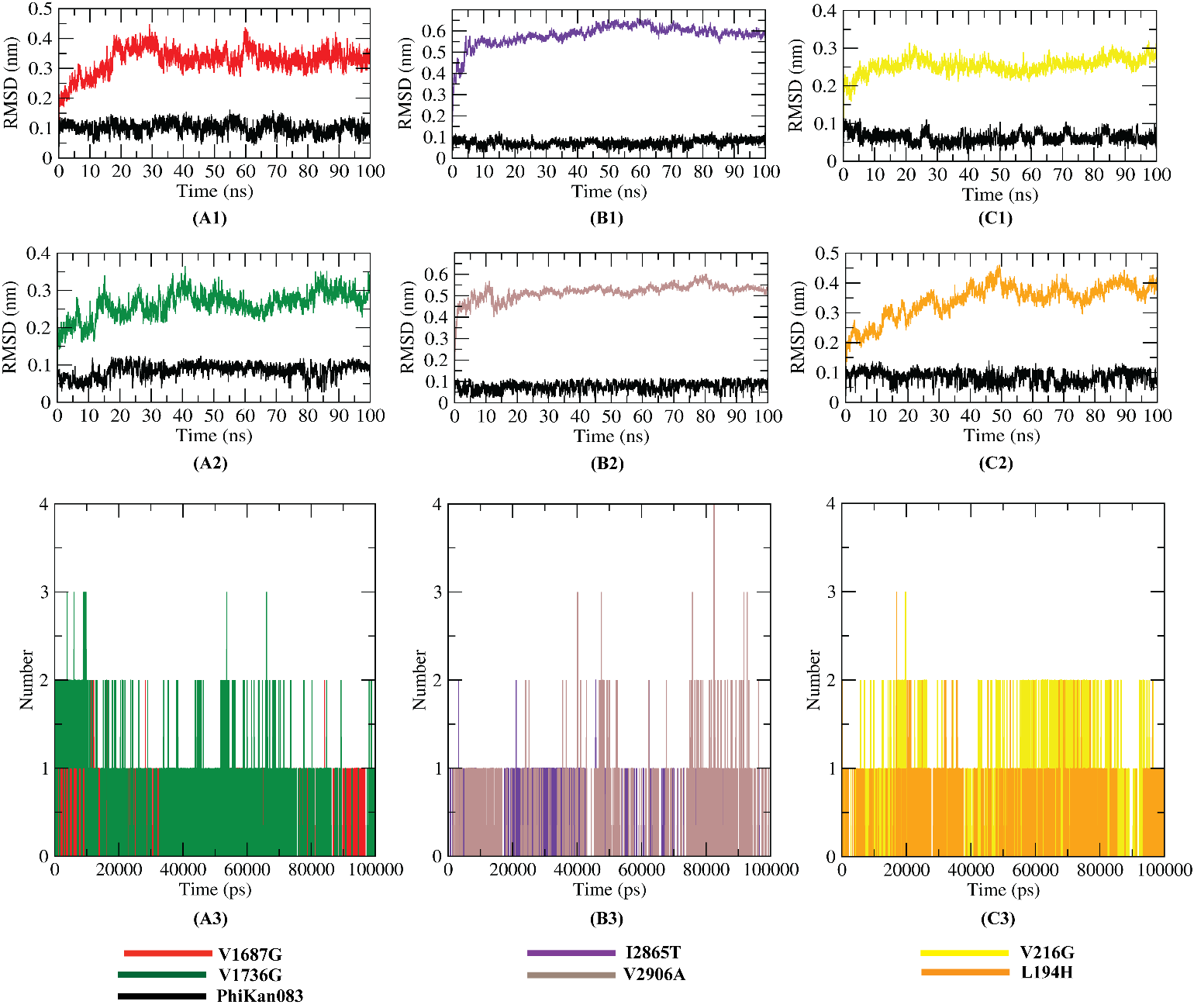


**Supplementary Figure S6:** MD simulation results (3^rd^ run) of mutant-PK083 complexes. (A1-C2) RMSD of PK083 and protein backbone obtained from 100 ns MD simulation. (A3-C3) Hydrogen bond analysis between PK083 and the mutants.
